# Caspofungin-Based Red-Emissive Probes for Fluorescent Imaging of Pathogenic Fungi

**DOI:** 10.1021/jacsau.6c00209

**Published:** 2026-05-13

**Authors:** Maxime Klausen, Richa Sharma, Jason L. Brown, Gordon Ramage, Mark Bradley

**Affiliations:** † Chimie ParisTech, PSL University, CNRS, Institute of Chemistry for Life and Health Sciences, Paris 75005, France; ‡ School of Chemistry, King’s Buildings, 3124 University of Edinburgh, Edinburgh EH9 3FJ, U.K.; § Glasgow Dental School and Hospital, Oral Sciences Research Group, School of Medicine, Dentistry and Nursing, 3526 University of Glasgow, Glasgow G2 3JZ, U.K.; ∥ Research Centre for Health, School of Health and Life Sciences, 3525 Glasgow Caledonian University, Glasgow G4 0BA, U.K.; ⊥ Precision Healthcare University Research Institute, Queen Mary University of London, Empire House, London E1 1HH, U.K.

**Keywords:** fluorescent imaging, echinocandins, caspofungin, fungal diagnostics, infectious diseases

## Abstract

Fungal infections are an often-neglected cause of morbidity worldwide, while their inaccurate and often inconclusive diagnosis indirectly promotes high morbidity and the development of antimicrobial resistance. Here, the clinically used semisynthetic echinocandin antifungal caspofungin was selectively conjugated to red-emitting fluorescent dyes to develop new optical imaging probes for fungal detection. The probes were characterized using established 2D NMR techniques to determine the specific position of the fluorophore on the peptide backbone, and both isomers demonstrated selective labeling of pathogenic fungal strains with similar efficacy. Selective multiplexed imaging of target species was achieved in a co-culture of fungi and bacteria, including when using benchtop imaging platforms.

## Graphical abstract

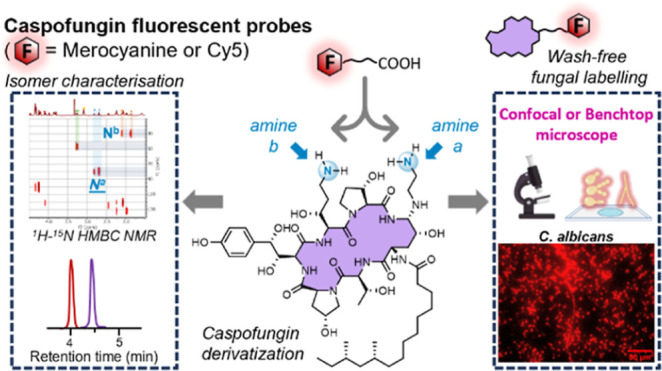

Fungal infections pose a severe threat to human health with over 150 million cases worldwide and 1.7 million deaths per annum.^1^ The global burden of fungal infections has become increasingly alarming over the past decades, exacerbated by a warmer climate, fungicidal (over)­use in agriculture, and the COVID-19 pandemic, which led to a sharp decline of testing for pathogens, strained healthcare infrastructures, and increased use of corticosteroids.^2,3^ This has driven the emergence of multidrug-resistant fungi as well as previously unknown and potentially life-threatening pathogens.^4^ This threat was underscored by the WHO in 2022 with the release of its first list of fungal pathogens associated with a serious risk of mortality or morbidity, in which *Candida albicans* and *Candida auris* were designated as critical. *C. auris*, a yeast pathogen first described in 2009, exemplifies the severity of the crisis with intrinsic fluconazole resistance and rapidly evolving cross-resistance to echinocandins and polyenes across six continents.^5^

However, the fungal threat often remains underlying and neglected because of inefficient, insensitive, and time-consuming diagnostic techniques, leading to a large number of cases remaining undetected.^6^ Typical diagnostic practice involves collecting samples from the patient, followed by microbial culture and subsequent characterization and pathogen identification.^7^ However, conventional fungal cultures often require several weeks, such that timely and appropriate antimicrobial therapy is delayed. This not only increases mortality rates but also encourages precautionary empirical antifungal administration that promotes the rapid development of resistance mechanisms in these fungal pathogens.^8,9^ Alternative characterization techniques such as nucleic acid–based diagnosis are expensive, require dedicated instruments, trained personnel, and lengthy sample processing procedures. For low-resource areas/countries, this leads to no, failed, or delayed diagnosis in environments where disease burden is the heaviest.^10^ Thus, there is an urgent need to direct the diagnosis toward precise, rapid, and simpler techniques.^11^

Labeling pathogens with specific fluorescent probes can be an effective tool to rapidly identify specific infectious agents, allowing early treatment and prevention of increases in disease severity and fatality. Indeed, fluorescent probes have been increasingly used to identify/detect Gram positive,^12–14^ Gram negative bacteria,^15–17^ and mycobacteria.^18–20^ However, probes targeting fungal infections remain much less investigated.^21–25^ The development of pathogen-specific fluorescent probe is even more critical considering that infections caused by fungal pathogens are often mixed together with bacterial species, including in biofilms.^26^

Toward this goal, we report the design of fungal-specific fluorescent probes by selectively conjugating far-red emitting dyes to one of the two amine groups on the antifungal lipopeptide agent caspofungin (Figure 1). Caspofungin is a semisynthetic drug prepared from the natural echinocandin derivative pneumocandin and has become a popular antifungal agent because of its specific mode of action, broad-spectrum activity, and low systemic toxicity.^27,28^ Caspofungin inhibits the subunit FKS1 of 1,3-β-glucan synthase (1,3-β-GS), an enzyme complex composed of subunits FKS1, FKS2, and rho that are found only in fungi.^29^ Glucan synthase is essential for the dynamic formation of the fungal cell wall and cell division, and its inhibition arrests fungal growth. Caspofungin inhibits this membrane bound enzyme without requiring internalization into the cell cytoplasm (the lipopeptide’s hydrophobic alkyl tail intercalates into the membrane bilayer through a passive uptake process and is then positioned locally to allow proximal blocking of synthase activity), with the echinocandin binding site sitting in a hydrophobic interfacial pocket in close contact with the membrane. Hence, an optical probe derived from this lipopeptide would be expected to preferentially target fungi while minimizing labeling of bacterial and mammalian cells, thereby providing selectivity for use in diagnostics.^24^

**Figure 1:**
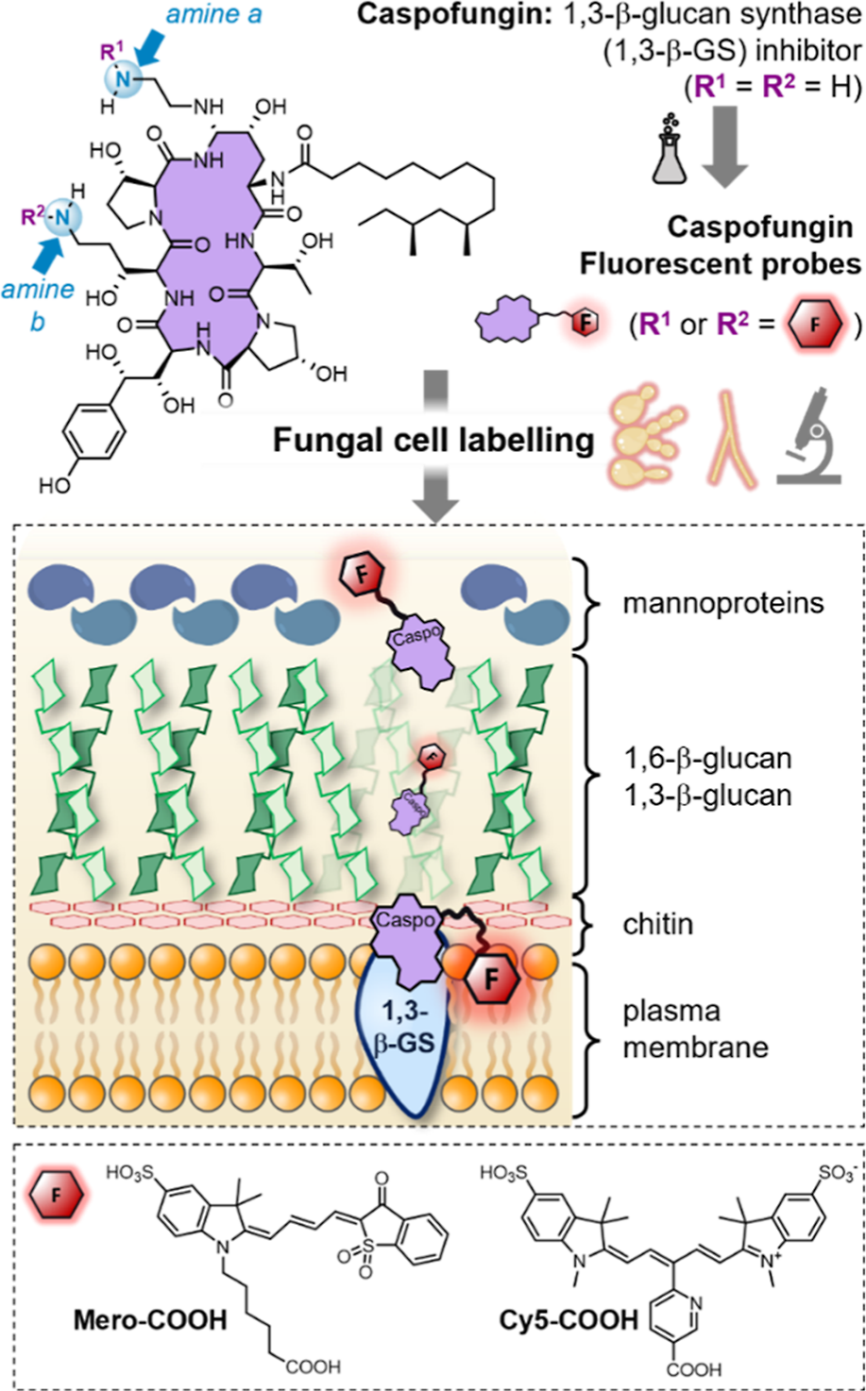
General overview of the fungal-labeling strategy and their proposed mechanism of action. The caspofungin lipopeptide (Caspo, purple) is a known binder of the transmembrane protein, 1,3-β-glucan synthase (light blue), and is therefore expected to enable selective fluorescent labeling of pathogenic fungi when used as a probe targeting scaffold.

Thus, caspofungin has become an attractive targeting ligand in molecular diagnostics, notably through direct conjugation of green-emitting BODIPY^25^ and red-emitting 7-amino-9H-(1,3-dichloro-9,9-dimethylacridin-2-one) (DDAO)^22^ dyes to the readily accessible amines on the cyclic peptide (hereafter referred to as N^a^ and N^b^) by one-step amide coupling. However, this direct but uncontrolled derivatization strategy will intrinsically generate a mixture of fluorescent isomers (A and B), which are susceptible to differences in binding affinity through loss of an amine group and dye attachment. Other strategies have been explored to circumvent this regioselectivity challenge, such as the modification of the homotyrosine moiety with a click chemistry handle for dye attachment.^30^ However, while elegant, this approach required additional synthetic steps on a key echinocandin residue, making it less synthetically accessible compared to one-step peptide derivatization by amide coupling. Consequently, although N^a^ and N^b^ represent the most convenient handles for fungal probe synthesis, the fundamental challenge associated with the selectivity and performance of the A and B amide derivatives of caspofungin remains unanswered. In this work, we investigate the potential of direct derivatization of caspofungin as a diagnostic imaging approach by developing red-emissive, fungal-specific caspofungin probes combining (i) selective identification of clinically relevant fungal strains with affordable imaging equipment (a crucial point for diagnostic use in low-income settings), and (ii) individual A/B isomer identification and comparison for potential differences in labeling that would indicate whether site-selective derivatization strategies should be preferred. To address the first point, we used a complementary pair of fluorophores for caspofungin derivatization: an “always-on” sulfonated Cyanine 5 (Cy5, λex/em = 650/671 nm) and an environmentally responsive sulfonated merocyanine (Mero, λex/em = 597/627 nm). Their robust red emission profiles mitigate sample autofluorescence and background interference, particularly strong for fungi under green illumination. Importantly, solvato-fluorogenic merocyanines^31^ are “switched-on” in hydrophobic environments, which makes them particularly suitable for probes binding into hydrophobic cell membrane environments, where the brightness increase removes the need to wash away unbound dye and enables clinical/in vivo imaging. To investigate the selectivity of the N^a^/N^b^ functionalization of Capsofungin and the resulting effect on probe binding (Figure 1), the probes were synthesized by following two complementary pathways allowing the separation and identification of each positional isomer. Structural confirmation was unequivocally obtained using 2D ^1^H and ^15^N NMR techniques, and the isomers were then tested, both individually and as a mixture, in fungal-labeling experiments to determine the performance of these probes as diagnostic tools.

The design of our fluorescent probes was based on amide coupling between one of the two primary amines N^a^ and N^b^ of caspofungin and the carboxylic acid of the two red-emissive dyes (Scheme 1). Detailed synthesis procedures and characterization are given in the ESI. **Mero-COOH** is a push–pull merocyanine dye^31^ constituted of an indoline electron-donating group and a benzothiophenone-1,1-dioxide electron-withdrawing moiety. The sulfonated indoline was alkylated with 6-bromohexanoic acid to introduce a carboxylic acid group, which allows conjugation onto the amine groups of caspofungin. This C-6 alkyl spacer enhances the spatial separation between the antifungal agent and the fluorophore, thereby minimizing the potential negative effects of introducing a relatively bulky fluorophore on the binding of the probe to caspofungin. The synthesis of the dye was achieved in 2 steps by following known procedures.^31^ Bis-sulfonated cyanine dye **Cy5-COOH** was synthesized by condensation between the corresponding sulfonated indolium and carboxylated pyridine malondialdehyde as previously reported.^32,33^ This provided a water-soluble bis-sulfonated cyanine with an aromatic spacer.

**Scheme 1:**
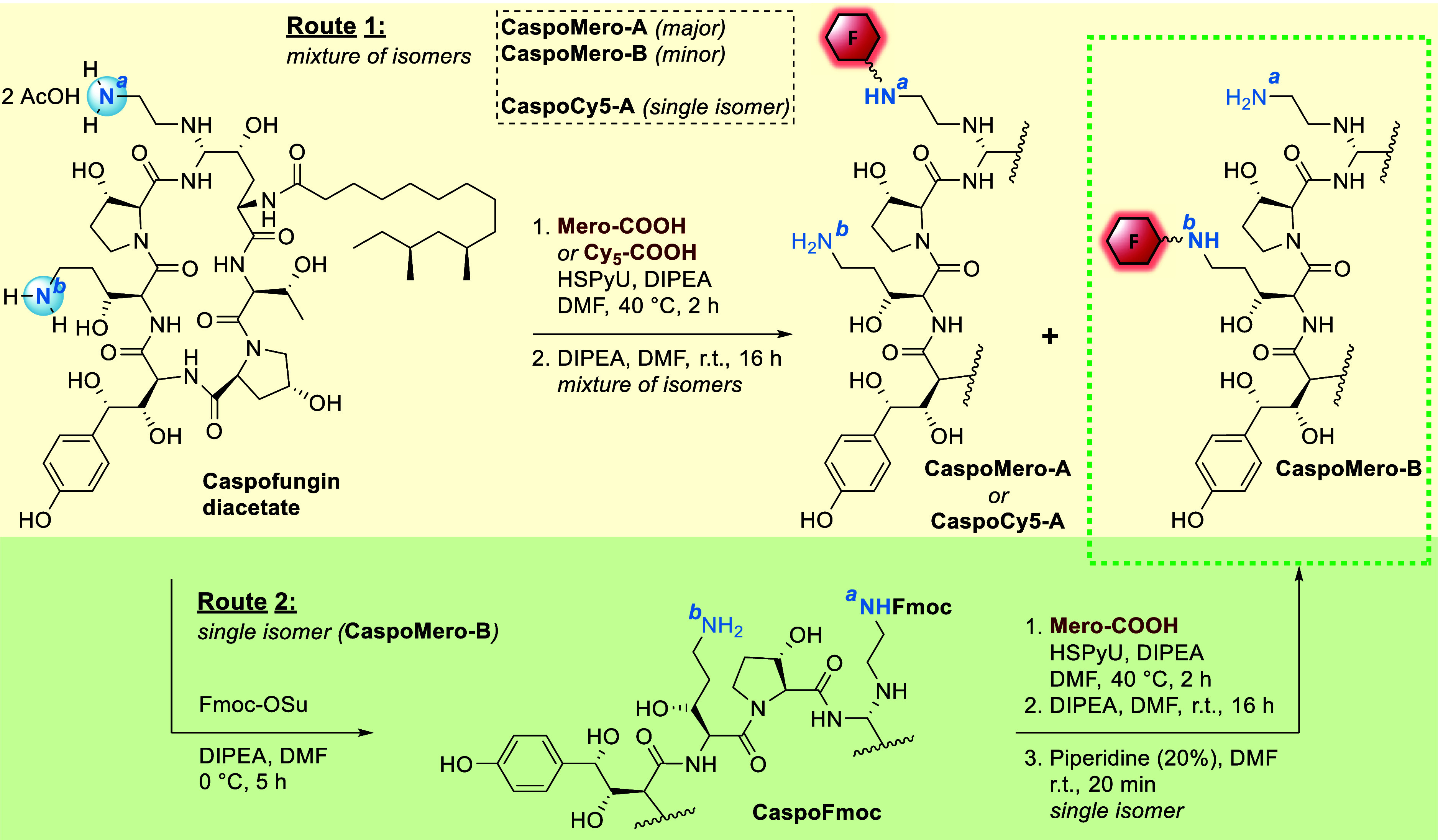
Synthetic Pathways for the Conjugation of Fluorophores **Mero** and **Cy5** onto Caspofungin^a^

a Route 1 (top) leads primarily to conjugation on amine N^a^, while route 2 (bottom) leads to dye conjugation on amine N^b^ via a selective Fmoc protection strategy.
b All probes were characterized by HPLC and HRMS (see ESI).

Dye conjugation was initially performed by direct amide coupling on commercially available caspofungin diacetate (Scheme 1, route 1). To this end, both dyes **Mero-COOH** and **Cy5-COOH** were activated with the coupling agent HSPyU, monitoring the conversion to the *N*-hydroxysuccinimide ester by LC–MS. The active ester was then reacted with caspofungin. Unsurprisingly, reaction with fluorophore **Mero-COOH** led to a mixture of position isomers **CaspoMero-A** and **CaspoMero-B** with similar MS spectra (see ESI) due to the conjugation occurring on each of the two terminal amines. The isomers were effectively separated by preparative HPLC, in a ratio of 4:1. With reports indicating that the amine N^a^ of caspofungin would typically be the more reactive in amide coupling reactions,^34^ the major isomer was hypothesized to be **CaspoMero-A**.

Interestingly, reaction with bis-sulfonated cyanine **Cy5-COOH** led to the formation of a single isomer as observed by LC–MS, likely also resulting from coupling on amine N^a^. The selective formation of **CaspoCy5-A** presumably results from the higher steric hindrance and lower reactivity of the aromatic carboxylic acid of **Cy5-COOH**, preventing coupling on the less reactive amine N^b^. Conversely, the longer and more flexible spacer on **Mero-COOH** would allow for attachment to both amines.

To formally identify the point of attachment of the dyes in our fungal probes, we combined chemical and analytical techniques. Thus, a complementary synthetic route generating preferentially the minor isomer was explored (Scheme 1, route 2). Caspofungin was initially reacted with 1 equivalent of Fmoc *N*-hydroxysuccinimide ester at low temperature, leading to selective protection of amine N^a^.^34,35^ The intermediate (**Fmoc-N** ^**a**^-**Caspo**) was isolated and fully characterized (see below and ESI) and then reacted with the NHS active ester of **Mero-COOH**. Fmoc deprotection and subsequent purification gave a fluorescent product with an identical HPLC retention time as the minor isomer isolated from route 1 (*t*_R_ = 4.58 min, see the ESI), confirming that **CaspoMero-B** is generated via conjugation at the N^b^ position. As confirmation of this strategy, performing this Fmoc protection/coupling/deprotection route with the bis-sulfonated cyanine dye **Cy5-COOH** indeed provided a different HPLC trace from **CaspoCy5-A**, attributed to the complementary isomer **CaspoCy5-B** generated as an analytical sample (see the ESI). Probes **CaspoMero-A, -B** and **CaspoCy5-A** were further characterized structurally and biologically to elucidate differences between isomers and fungal-labeling efficiency.

The successful attachment of the dyes on probes **CaspoMero-A** and **CaspoCy5-A** was clearly evidenced by ^1^H NMR, with key aromatic signals characteristic of the merocyanine and cyanine dyes, respectively, and singlets associated with their dimethylindole moieties identifiable on their spectra (see the ESI). To clearly identify the attachment site of our dyes on caspofungin and validate the selectivity of the amide coupling reaction, ^1^H and ^15^N NMR techniques (1D and 2D) were used to unravel the fingerprint of unmodified caspofungin and compare it with **Fmoc-N** ^**a**^-**Caspo** (Figure 2, ESI Table S1). Long-range ^1^H–^15^N HMBC, an established NMR technique circumventing the low natural abundance of ^15^N NMR, proved particularly instrumental in isomer assignment.

**Figure 2:**
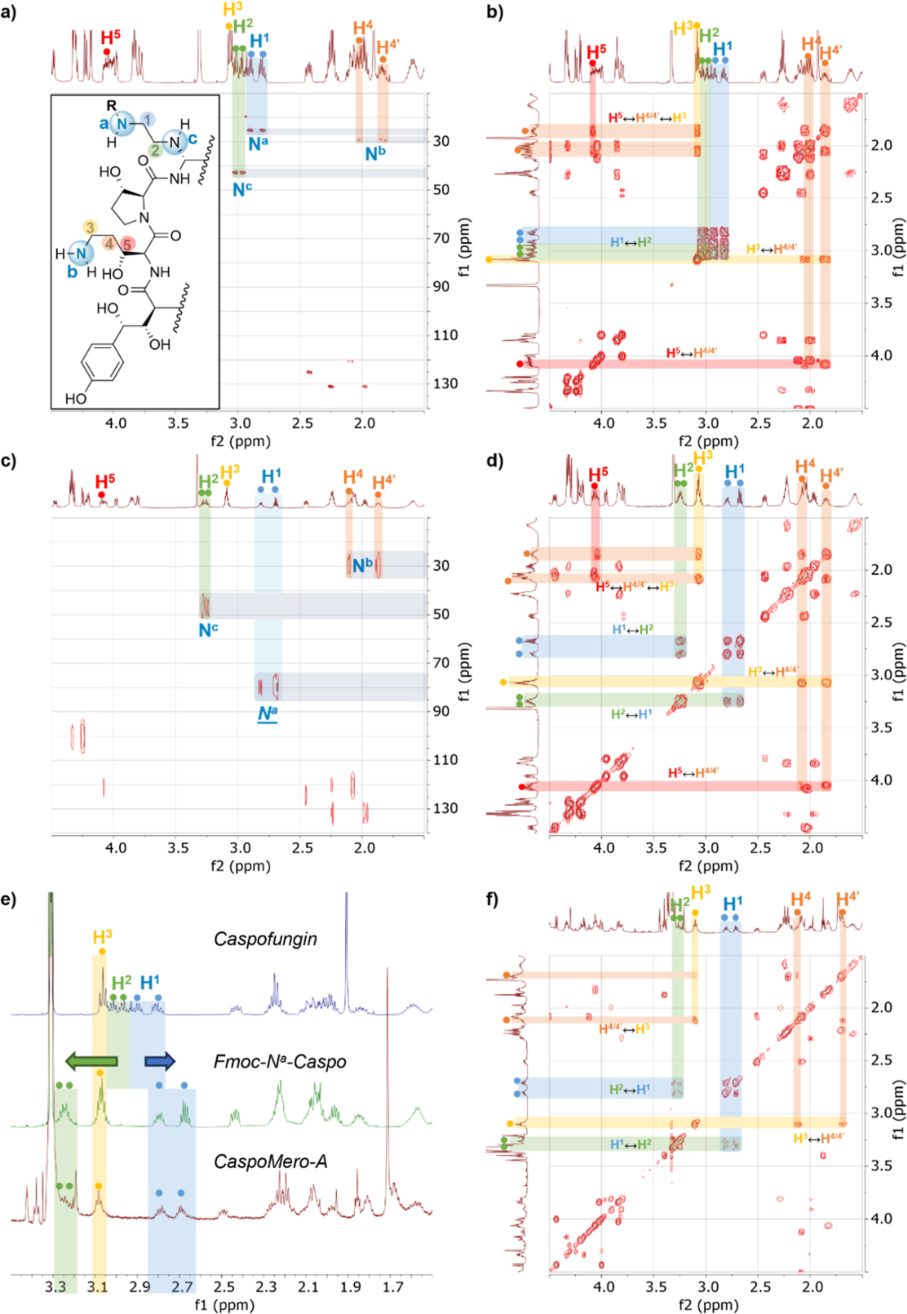
NMR spectra of caspofungin and N^a^-functionalized derivatives (all in CD_3_OD). (a) ^1^H–^15^N- HMBC spectrum and (b) ^1^H–^1^H COSY spectrum of caspofungin. (c) ^1^H–^15^N HMBC spectrum and (d) ^1^H–^1^H COSY spectrum of Fmoc-N^a^-Caspo. (e) Stacked ^1^H NMR spectra of caspofungin (top), Fmoc-N^a^-Caspo (middle), and CaspoMero-A (bottom), highlighting the shift of signals H^1^ and H^2^ upon functionalization of amine N^a^, while signal H^3^ remains untouched. (f) ^1^H–^1^H COSY spectrum of CaspoMero-A. The insert on panel (a) shows the partial chemical structure of caspofungin (R = H), Fmoc-N^a^-Caspo (R = Fmoc), and CaspoMero-A (R = Mero dye), with labels used for NMR assignment.

The ^1^H NMR of caspofungin showed two clearly identifiable doublets in the aromatic region, corresponding to the phenol moiety, followed by a succession of signals between 5.5 and 1.5 ppm corresponding to the alpha protons of the backbone and side chains of the cyclic peptide. To identify the signals corresponding to the two key amino groups, we correlated this data with a ^15^N HMBC spectrum. The ^15^N signals were divided into two areas corresponding to aliphatic amines (10–50 ppm) and amides (80–140 ppm) (Figure 2a). The two upfield signals (25.3 and 29.4 ppm) can be attributed respectively to primary amines N^a^ and N^b^, while the secondary amine (hereby labeled N^c^) is slightly unshielded at 42.8 ppm. Sequential through–bond correlation between N^a^, H^1^, H^2^ and N^c^ was confirmed by combined analysis of the ^15^N HMBC and COSY NMRs (Figure 2a,b). This assigned the ^1^H NMR multiplets at 2.94–2.76 and 3.03–2.94 ppm respectively to H^1^ and H^2^, as the only proton signals correlating together and being enclosed between two nitrogens. The remaining ^15^N signal at 29.4 ppm, can thus be attributed to N^b^, and correlated with two neighboring multiplets at 2.02–1.93 and 1.89–1.77. These signals located in the aliphatic region were attributed to the diastereotopic protons H^4^ and H^4^’, coupling with N^b^ (via a ^3^ *J*), and with unshielded multiplets at 3.09–3.03 and 4.05 ppm. Although a direct ^2^ *J* correlation with the N^b^ signal was not observed, ^1^H chemical shifts unambiguously indicated that the former multiplet corresponds to H^3^, and the latter to H^5^, as explained by the presence of the neighboring hydroxyl group. This data is in accordance with the reported assignments for caspofungin.^35^

The NMR data for **Fmoc-N** ^**a**^-**Caspo** were compared with this assignment. As expected, only two signals remained in the aliphatic amine region of the ^15^N HMBC spectrum (Figure 2c), at 29.5 ppm (N^b^) and 47.0 ppm (N^c^), which were almost unaffected by Fmoc protection and retained similar chemical shifts. Conversely, a significant downfield shift of the ^15^N signal for N^a^ to 79.8 ppm directly reflects conversion of this primary amine to a carbamate, thereby validating selective Fmoc protection and supporting the assignment of **CaspoMero-B** as the N^b^ isomer. A slight deshielding was also observed for N^c^, which could be related to the effect of the carbonate of the nearby Fmoc group. Thus, it is also possible to attribute the ^1^H signal at 2.73 ppm to H^1^. Although no through–space correlations could be evidenced between the Fmoc signals and the aliphatic linker signals on the ROESY spectrum (Figure S3), correlations could be sequentially confirmed through bonds and through space with COSY (Figure 2d) and ROESY experiments. Other key proton signals remained in their original range (Table S1), further confirming these assignments.

Comparison of the ^1^H NMR signals in the 1.5–4.5 ppm region of interest of the starting caspofungin material, the Fmoc protected caspofungin and **CaspoMero-A** showed an identical pattern after functionalization (Figure 2e). Through bond correlations observed in the ^1^H–^1^H COSY NMR of **CaspoMero-A** (Figure 2f) directly validated that the H^1^ signal was shifted to 2.84–2.64 ppm following amide bond formation with the merocyanine. Conversely, the H^3^ signal remained in its original range indicating no functionalization of neighboring amine N^b^, as observed after the Fmoc protection of caspofungin. Similar signals and coupling patterns between H^1^–H^2^ and H^3^–H^^4^/^4^’^ unequivocally evidenced the position of the dye in **CaspoMero-B.**

The same pattern was observed on an NMR of **CaspoCy5-A** (ESI, Figure S4), consistently confirming preferential amide coupling with amine N^a^. Therefore, with NMR evidence of selective Fmoc protection of amine N^a^ and with HPLC retention times and MS data obtained on the probes, we obtained conclusive confirmation that **CaspoMero-B** bears the fluorophore on amine N^b^ and is also the minor product of synthetic route 1.

The optical properties of the probes **CaspoMero-A**, with a solvato-fluorogenic fluorophore, and **CaspoCy5-A**, with a sulfonated (always on) cyanine dye, were investigated in DMSO (Figure 3). The properties of the caspofungin-probe conjugates were essentially comparable to those of the parent dyes in solution. In DMSO, **CaspoMero-A** showed an absorption band peaking at 593 nm and a strong emission at 628 nm (Table 1). The absorption and fluorescence of **CaspoCy5-A** were both markedly red-shifted in comparison, with peaks, respectively, at 650 and 671 nm and extending into the near-infrared. The Stokes shift value for the merocyanine probe was double that of the Cy5 probe, which is consistent with the properties of the isolated dyes. Both compounds showed significant brightness values, with the cyanine probe showing a 3-fold higher brightness compared to the merocyanine derivative because of its higher absorption coefficient and quantum yield. The emission properties of isomers **CaspoMero-A** and **CaspoMero-B** showed no significant deviations (ESI Figure S5), as their emission profiles perfectly overlapped with only minor brightness differences.

**Figure 3:**
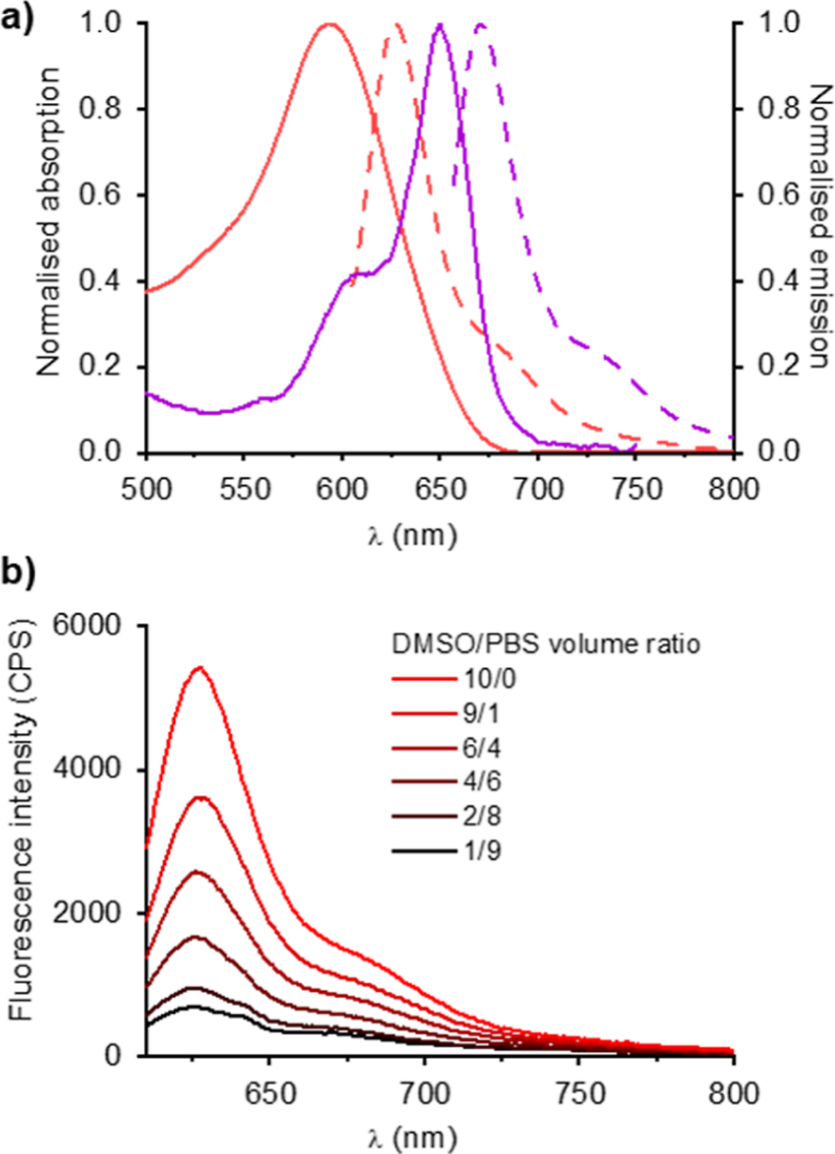
(a) Normalized absorption (solid line) and normalized emission spectra (dashed line) of CaspoMero-A (red) and CaspoCy5-A (purple) in DMSO. (b) Evolution of the fluorescence spectra of CaspoMero-A with increasing percentages of PBS in DMSO.

**Table 1:** Comparison of the Photophysical Properties of “Environmental” Probe CaspoMero-A and “Always-On” Probes CaspoCy5-A in DMSO

Compound	λ_abs_^max^ (nm)	ε^max^ (M^–1^ cm^–1^)	λ_em_^max^ (nm)	Stokes Shift (cm^–1^)	Φ_f_	ε^max^Φ_f_ (M^–1^ cm^–1^)
CaspoMero-A	593	1.5 × 10^4^	628	940	0.25^a^	3.8 × 10^3^
CaspoCy5-A	650	2.9 × 10^4^	671	481	0.45^b^	13 × 10^3^

a Fluorescence quantum yield standard: Rhodamine 6G in EtOH (Φ_f_ = 0.94).

b Fluorescence quantum yield standard: Cresyl Violet in MeOH (Φ_f_ = 0.54).

The fluorescence of merocyanine dyes is known to be highly sensitive to the microenvironment of the dye, and as such, fluorescence spectra were recorded in DMSO with gradually increased levels of PBS. As expected, these showed a significant decrease in fluorescence with increased water (PBS) content (Figure 3). The 8-fold increase in fluorescence observed in hydrophobic environments indicates the probe can switch on in membranes with low background in aqueous media. Echinocandin drugs like caspofungin are found to be embedded and anchored in the plasma membrane via their hydrophobic tail when they bind to the FKS1 subunit of 1,3-β-GS.^29^ Therefore, it can be hypothesized that **CaspoMero-A** would label the target pathogens efficiently when anchored in the membrane and/or trafficked into vacuoles through endocytosis,^30^ without need to wash away any excess probe.

The distinguishing feature of these fungal probes is a combination of a strong optical signature and expected fungal targeting through the known echinocandin pathways, including 1,3-β-GS. The primary goal was therefore to verify that these properties translate into sensitive and specific labeling of fungi. The first aim was to determine the labeling capabilities and minimal concentrations of the probes required to visualize individual fungal cells, with *C. albicans*selected as a model organism.

A fixed number of fungal cells were immobilized onto poly d-lysine coated wells of Ibidi μ-Slides and incubated with probes **CaspoMero-A**, **-** **B** and **CaspoCy5-A** (0.1 to 50 μM) for 2 h without washing away the excess of probe. Both isomers had comparable labeling efficiency (Figure 4c, ESI Figure S6b–d). Using a confocal setup, labeling with 0.1 and 0.5 μM was negligible, whereas 2 μM provided robust labeling of all fungal cells for all probes (Figure 4). Hence, 2 μM was selected for subsequent experiments which also translates into frugal amounts of the probes. To establish the potential of the Caspo probes as possible clinical diagnostic tools in limited resource settings, we investigated their versatility and specificity using a nonconfocal plug-and-play EVOS M5000 benchtop microscope (Figure 5a) equipped with an LED light cube. Despite occasional higher background due to the benchtop instrument’s hardware (bandwidth, excitation tails), individual fungi were clearly seen without background fluorescence in wash-free samples, validating that merocyanine fluorescence was markedly enhanced in hydrophobic membrane-like environments. We quantified the fluorescence fold-increase, as well as analytical performance metrics, for *C. albicans* labeling using standard calibration-curve definitions (see ESI). At the selected standard working concentration (2 μM), the increase in fluorescence of treated cells vs untreated cells reached >1400-fold for both of our fluorogenic merocyanine probes, and only 9-fold for **CaspoCy5-A** (ESI, Table S2). Since, our total cell fluorescence values include background subtraction, these figures are consistent with the wash-free behavior of the solvato-fluorogenic dyes compared to the higher background for always-on Cy5 reporter, that results in lower relative fluorescence in treated cells at 2 μM. Within the initial linear segments of their response, both **CaspoMero-A** and **-B** had limits of detection (LODs) in the low nanomolar range (0.01 μM), and limits of quantification (LOQs) of 0.03–0.04 μM (ESI, Table S2, Figure S7). In contrast, **CaspoCy5-A** showed a LOD of 1.9 μM and a LOQ of 5.8 μM. The linear range of the Cy5 probe extended to >50 μM, while the environmental merocyanine probes showed logarithmic behavior above 2 μM (ESI, Figure S7), which further confirms their optical differences in cells.

**Figure 4:**
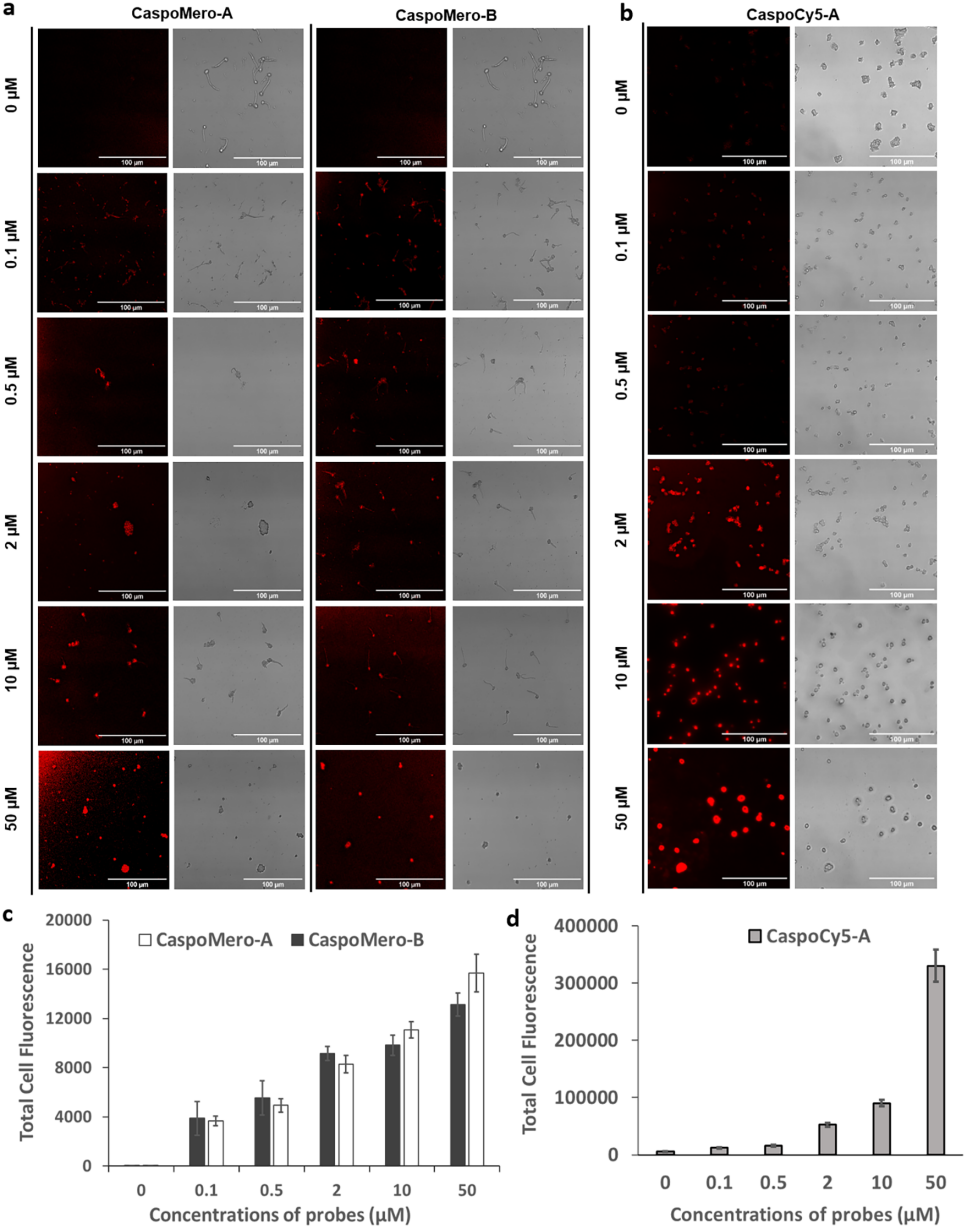
Wash-free labeling of *C. albicans* (10^5^ cfu/mL) immobilized onto poly d-lysine-coated wells of Ibidi μ-Slide 15-Well slides at different concentrations of (a) **CaspoMero-A** and **CaspoMero-B** (0–50 μM) with λex = 594 nm and λem = 630 nm, (b) **CaspoCy5-A** (0–50 μM) with λex = 650 nm and λem = 690 nm. Left column: fluorescent images, right column: brightfield images. Scale bars 100 μm. (c,d) Quantification of fluorescence intensity of probe binding. This was achieved using ImageJ (NIH) by measuring the mean intensity of 20 fluorescent spots and subtracting the mean of 10 background spots. Standard errors are represented by bars (*n* = 20). Images were obtained using a Leica TCS SP5 laser-scanning confocal microscope with TRITC (532–613 nm) and Cy5 (672–712 nm) detection.

**Figure 5:**
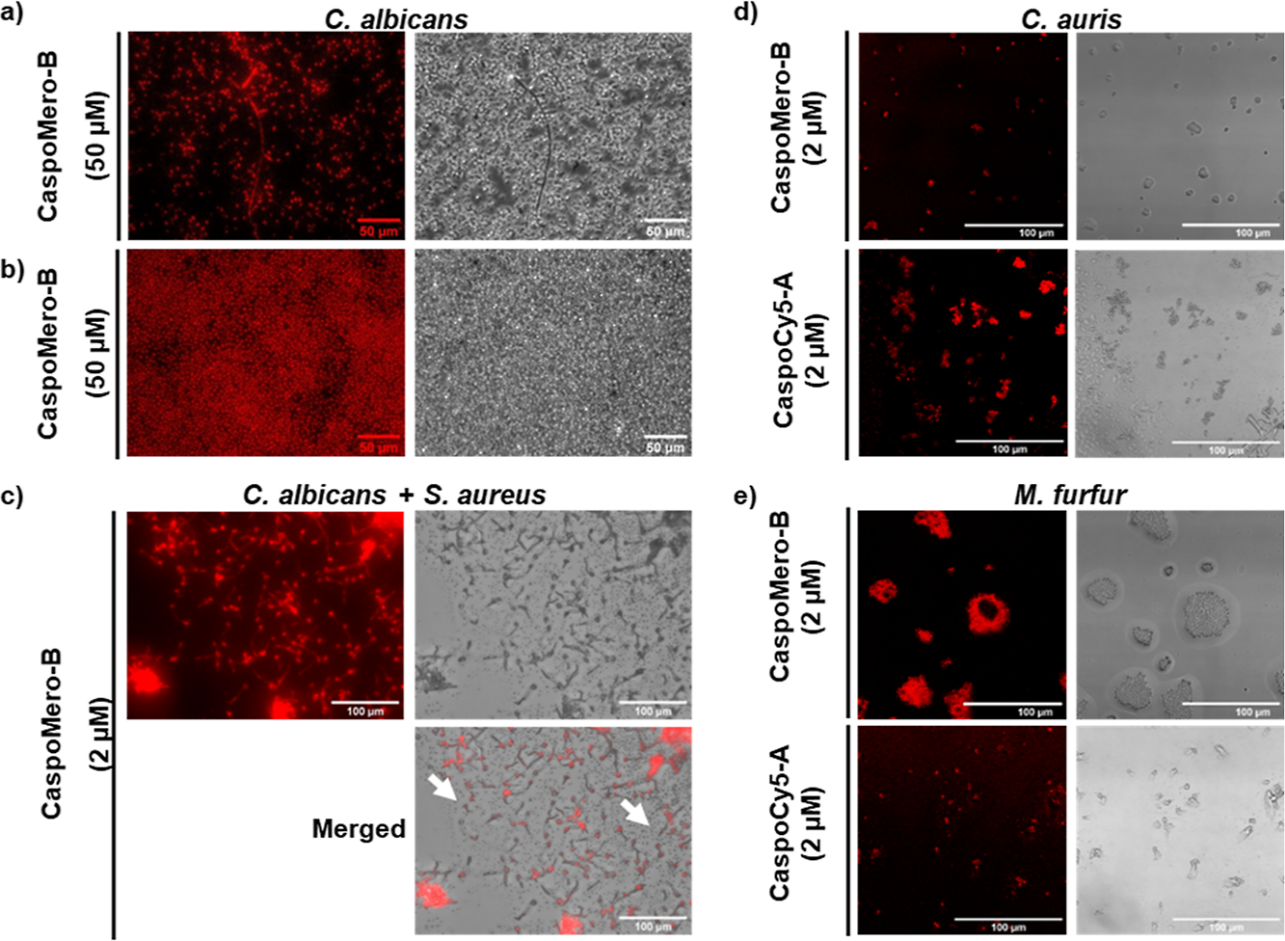
(a) Wash-free labeling of *C. albicans* pseudohyphae immobilized onto poly d-lysine coated wells of Ibidi μ-Slide 15-Well slides with **CaspoMero-B** (50 μM) using an EVOS M5000 benchtop microscope TRITC filter. Left column: fluorescent images, right column: brightfield images obtained with a transmitted light photomultiplier tube detector. Scale bars: 50 μm. (b) Fungal biofilms labeled by **CaspoMero-A** (50 μM) without wash step and visualized with an EVOS M5000 benchtop microscope TRITC filter. Scale bars: 50 μm. (c) **CaspoMero-B** (2 μM) specifically labeling fungal cells in a unwashed co-culture of *C. albicans* (10^5^ cfu/mL) and Gram-positive bacteria *Staphylococcus aureus* (10^8^ cfu/mL, visible as dots marked with white arrows) immobilized onto poly d-lysine coated wells of Ibidi μ-Slide 15-Well slides, visualized using an EVOS M5000 benchtop fluorescent microscope TRITC filter. Scale bars: 100 μm. (d) Wash-free labeling of clinically relevant pathogenic fungi *C. auris* and (e) *M. furfur* with **CaspoMero-B** (2 μM, λex = 594 nm and λem = 630 nm) and **CaspoCy5-A** (2 μM, λex = 650 nm and λem = 690 nm), imaged with a Leica TCS SP5 laser-scanning confocal microscope with TRITC (532–613 nm) and Cy5 (672–712 nm) detection. Scale bars: 100 μm. For each panel, left column: fluorescent images, right column: brightfield images obtained with a transmitted light photomultiplier tube detector.

The impact of fungal morphology on labeling efficiency was also investigated. Within the fungal population there are a number of short and long pseudohyphal structures which were distinctly labeled by the **CaspoMero** probe (Figure 5a), demonstrating that the presence of different fungal morphologies does not impede detection. This was also observed using the **CaspoCy5** probe under confocal imaging (ESI, Figure S8). We further aimed to simulate a key clinical challenge by testing the ability of our probes to penetrate and label fungal biofilms. Fungi often have a propensity to aggregate into biofilms, expressing a dense extracellular matrix in pathological settings that causes poor drug penetration and 100–1000-fold increased resistance to antifungals compared to planktonic cells, often leading to treatment failure.^36^ Thus, **CaspoMero-B** was incubated with 10^6^ cfu/mL *C. albicans* biofilms at 50 μM. Fluorescence images showed that the probe could efficiently permeate biofilms to label the embedded pathogen cells (Figure 5b). While conventional antifungal compounds require >100 times higher concentrations to penetrate high density cultures, the caspofungin probes effectively reached the embedded fungal cells and enabled visualization within the protective biofilm structure at 50 μM, which is a significant advantage as biofilm-associated infections are notoriously difficult to diagnose and treat.

Labeling selectivity at our standard working concentration against other common pathogens and mammalian cells was also investigated in this setting. To assess this, Gram negative (*Escherichia coli*) and positive (*Bacillus subtilis*, *Micrococcus luteus*) were incubated with **CaspoMero-A** and **-B** (5 μM), showing no fluorescence signal in wash-free conditions (ESI, Figure S9). A mixture of bacterial (*Staphylococcus aureus*) and fungal (*C. albicans*) cells were also incubated with the probes (2 μM) and imaged to confirm this selectivity in co-cultures. Excellent selectivity was observed for both **CaspoMero** (Figure 5c) and **CaspoCy5** probes (ESI, Figure S10), whereby only the fungal cells could be identified postlabeling and no fluorescence was detected from the bacteria. This selectivity is critical in a diagnostics context, as fungal and bacterial cells often coexist in clinical infections.

Interestingly, the versatility of the probes was further evidenced by co-labeling fungal cells with a molar ratio-adjusted mixed solution of either **CaspoMero-B** or **CaspoCy5-A** and a complementary green emissive amphotericin-based fungal probe AmB-PEG-NBD, previously described as a fungal membrane sterol stain^24^ (ESI, Figure S11). Good colocalization was observed between the two probes, expected to label via different binding pathways, particularly on the periphery of fungal cells. This confirms the orthogonality with other fungal imaging agents and expands the diagnostic toolbox for multiplexed pathogen imaging.

A crucial requirement for applications in vivo and in patient samples is probe specificity for pathogens against host cells—a parameter often overlooked in reports. Here, no labeling of HeLa cells was observed at and above our working probe concentration (5 μM) with **CaspoMero-A** or **-B**, which validates our design strategy and underscores the potential of caspofungin probes as a possible clinical diagnostic tool (ESI Figure S12). The absence of cross-labeling of nontarget mammalian and bacterial pathogen cells, combined with previous literature reports,^25,30^ suggest that membrane-specific interactions are important contributors of fungal-labeling under our imaging conditions. This is also supported by the retention of fluorescent labeling in fungi with gradually disintegrated envelopes (ESI, Figure S13), with signal reduction only observed at heavy disintegration stages.

Together, these observations are fully compatible with fungal membrane binding, in line with the expected membrane–proximal interaction described for echinocandin mode of action and the previously reported mechanism of fluorescent echinocandin derivatives.^30^ At the short incubation times used for imaging, the observed peripheral labeling of yeast and pseudohyphae suggests membrane-specific binding that does not strongly depend on energy-mediated uptake processes for imaging. However, additional probe- and time-dependent fungal membrane-specific interactions beyond direct FKS target engagement cannot be ruled out, including contributions of specific subcellular localization and metabolically driven pathways. The potential coexistence of these pathways could be confirmed with further mechanistic investigations, such as competitive target engagement assays and extended uptake studies, beyond the scope of this study.

Finally, applicability across different fungal species was assessed by testing the probes on additional clinically relevant strains by using a confocal microscope. The probes were found to label the dermatological pathogen *Malassezia furfur* (Figure 5d) and the emerging life-threatening pathogen *C. auris* (Figure 5e). This is significant as *C. auris* and *M. furfur* have different cell morphologies, and *C. auris* does not switch between yeast and hyphal forms like *C. albicans*, which validates wash-free labeling across fungal targets that present unique phenotypic structures.

In summary, using two complementary site-selective synthetic pathways, carboxyl-functionalized red-emitting fluorophores were successfully conjugated to each amine of the 1,3-β-GS inhibitor caspofungin, yielding a set of fungal-binding probes for rapid fluorescent pathogen detection. Established heteronuclear correlation NMR techniques were instrumental in providing definitive proof that the amide coupling reaction occurs preferentially on amine N^a^ of caspofungin. The isolated isomers showed only limited difference in optical properties and in fungal-labeling performance, showing that functionalization of either amine does not affect significantly the binding domain. Both the merocyanine and Cy5-based caspofungin probes demonstrated several crucial features in a challenging diagnostic landscape, including rapid pathogen detection (<2 h) at concentrations as low as 0.1 μM, and compatibility with standard fluorescence microscopy equipment. The use of a robust always-on dye and a solvato-fluorochromic dye enabled versatile imaging and confirmation of membrane binding. The order-of magnitude increase in fluorescence intensity observed for **CaspoMero** in hydrophobic environments not only allowed wash-free fungal imaging, but also suggests the probe experiences a hydrophobic microenvironment compatible with association at the membrane–protein interface and proximity of the lipid bilayer.^29^

The dye-derivatized probes retained high specificity with no cross-labeling observed in bacteria or mammalian cells. This is a crucial feature for fungal imaging as the high level of similarity between fungal and mammalian cell membranes makes membrane stains susceptible to cross-labeling,^37^ such as amphotericin B-based membrane-disrupting probes where cross-labeling can occur due to cholesterol binding in mammalian cells. Cell wall-specific fungal dyes targeting structural biomolecules like chitin may also lack specificity (e.g., calcofluor cross-labeling of elastin and collagen).^38^ Thus, targeting fungal-specific enzymes orthogonal to host tissues circumvents these issues.^39^ The specificity observed in our cross-labeling experiments hence validates the use of a 1,3-β-GS inhibitor as the fungal-targeting ligand for high specificity. The caspofungin probes displayed essential features to address the critical fungal diagnostic bottlenecks in clinics, where culture-based methods can be slow and inadequate^7^ and where limited resources or diagnostic capacity gaps lead to empiric treatment.^10^ First, these probes were able to label several strains of fungi, including clinically relevant species such as the emerging hospital pathogen *C. auris* and the dermatological pathogen *M. furfur*, demonstrating applicability across diverse fungal infections. Second, the probes successfully penetrated and labeled fungal biofilms, which is key for use in clinical settings where biofilms often represent an intractable therapeutic target and lead to high rates of treatment failure.^26^ The combination of rapid staining, high specificity, biofilm penetration, and compatibility with simple microscopes supports the potential of these probes as promising candidates for fast diagnosis of fungal infections even with limited equipment and trained personnel available.

The probes outperformed previously reported fungal imaging agents^24,25,30,40^ that are typically applied at much higher concentrations (e.g., 400 μM for fungal keratitis diagnosis with a caspofungin-DDAO conjugate)^22^ or lack comprehensive validation for diagnostic use (e.g., BODIPY, NBD, and TMR probes). Finally, we demonstrated that direct derivatization of caspofungin with carboxyl-functionalized dyes led preferentially to functionalization of amine N^a^, with limited differences on the binding efficiency observed between isomers A and B. This provides important insight on caspofungin-derived diagnostic tools, where direct nonspecific amide coupling onto the N^a^ and N^b^ primary-amine handles is an accessible and straightforward derivatization technique,^22^ and the resulting isomers and mixtures of isomers maintain similar labeling capacity. Recent studies suggest that echinocandin binding is primarily driven by lipid tail anchoring, while the N^a^ and N^b^ cationic groups participating in solubility and membrane accumulation with a good tolerance to modification across the family of echinocandin drug derivatives.^27,28^ ^,^ ^29^ This would be in line with the retention of fungal-binding capacity of both derivatized caspofungin isomer probes A and B, thereby making amide coupling a viable strategy for probe synthesis. The attachment of a solvato-fluorogenic dye^31^ also generated probes with high sensitivity and high potential for clinical fungal diagnostics. The present work therefore provides a regioisomer-resolved approach toward quantitatively benchmarked caspofungin probes for wash-free fluorogenic fungal diagnostics, enabling visualization of clinically relevant strains on widely available benchtop microscopes. Future work will involve mechanistic studies to further assess target engagement in caspofungin probes and understand the impact of amine functionalization on receptor binding and uptake. Validation across a wider panel of target and nontarget cells, including with patient samples, will also be performed to drive our diagnostic approach toward clinical translation.

## Supplementary Material



## Notes

Key NMR signal assignments, imaging performance metrics, probe analytical data (HPLC, NMR, and fluorescence), additional fluorescence microscopy images and related quantification, additional NMR spectra, HPLC, MS data and details on experimental procedures, and materials and methods (PDF)
