# Supplementary material for: Caspofungin-Based Red-Emissive Probes for Fluorescent Imaging of Pathogenic Fungi
Source: JACS Au. 2026 May 13;6(5):2881–90. doi: 10.1021/jacsau.6c00209 (PMC13213487; doi:10.1021/jacsau.6c00209)
Supplement: Supplementary file 1 [file au6c00209_si_001.pdf]

Caspofungin-based red-emissive probes for fluorescent imaging of pathogenic fungi

Maxime Klausen,*#a Richa Sharma,#b Jason L. Brown,c Gordon Ramage,c,d* Mark Bradleye*

a. Chimie ParisTech, PSL University, CNRS, Institute of Chemistry for Life and Health Sciences, 75005 Paris, France. Email: maxime.klausen@chimieparistech.psl.eu
b. School of Chemistry, King's Buildings, University of Edinburgh, Edinburgh, EH9 3FJ, UK.
c. Glasgow Dental School and Hospital, Oral Sciences Research Group, School of Medicine, Dentistry and Nursing, University of Glasgow, G2 3JZ, UK.
d. Research Centre for Health, School of Health and Life Sciences, Glasgow Caledonian University, Glasgow, UK. Email: Gordon.ramage@gcu.ac.uk
e. Precision Healthcare University Research Institute, Queen Mary University of London, Empire House, London, E1 1HH. Email: m.bradley@qmul.ac.uk
# Authors contributed equally to this work.

SUPPORTING INFORMATION

Table of content

I. Supporting Figures, Schemes and Tables 2
II. Chemical synthesis 10
1. Materials and methods 10
2. Synthetic procedures and characterisation 11
III. Photophysical experiments 13
IV. Biology 14
V. NMR, MS, and HPLC Data 17
VI. References 23

I. Supporting Figures, Schemes and Tables

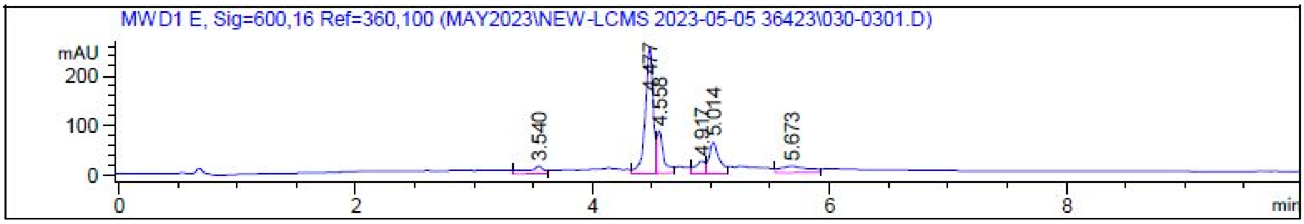

Figure S1. HPLC trace (600 nm detection) of the crude mixture of **CaspoMero-A** (t_R = 4.477 min) and **-B** (t_R = 4.558 min) obtained by following synthesis path A. An 8:2 ratio was obtained for products generated by reaction at the amine sites N^a:N^b following purification by Prep RP-HPLC.

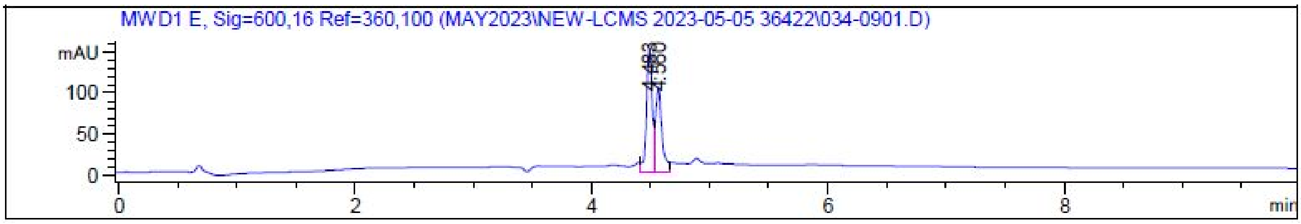

Figure S2. HPLC trace (600 nm detection) of a mixture of **CaspoMero-A** and **-B** obtained during purification on Prep-HPLC.

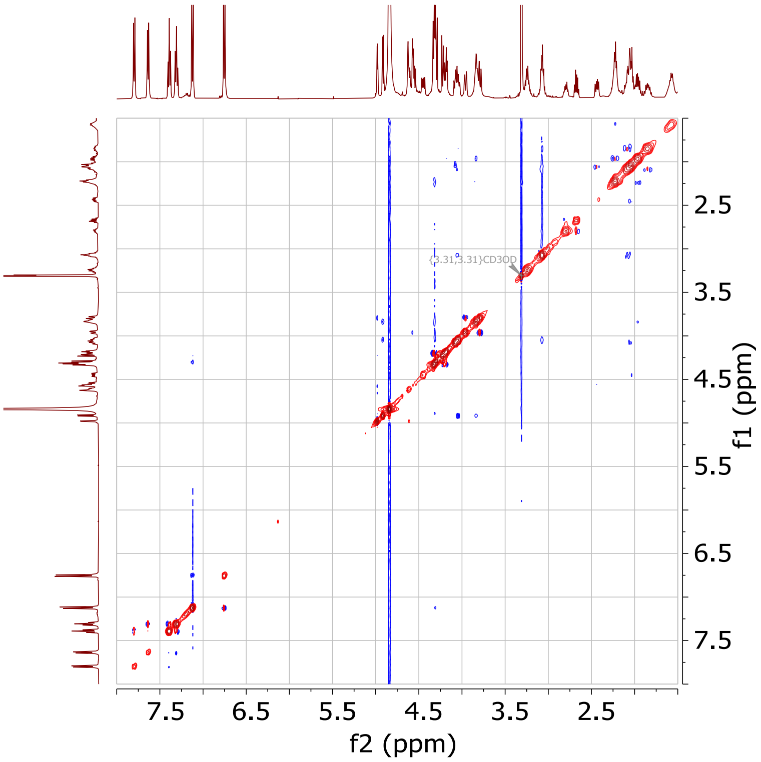

Figure S3. ^1H-^1H ROESY spectrum of **Fmoc-N^a-Caspo** in CD_3OD.

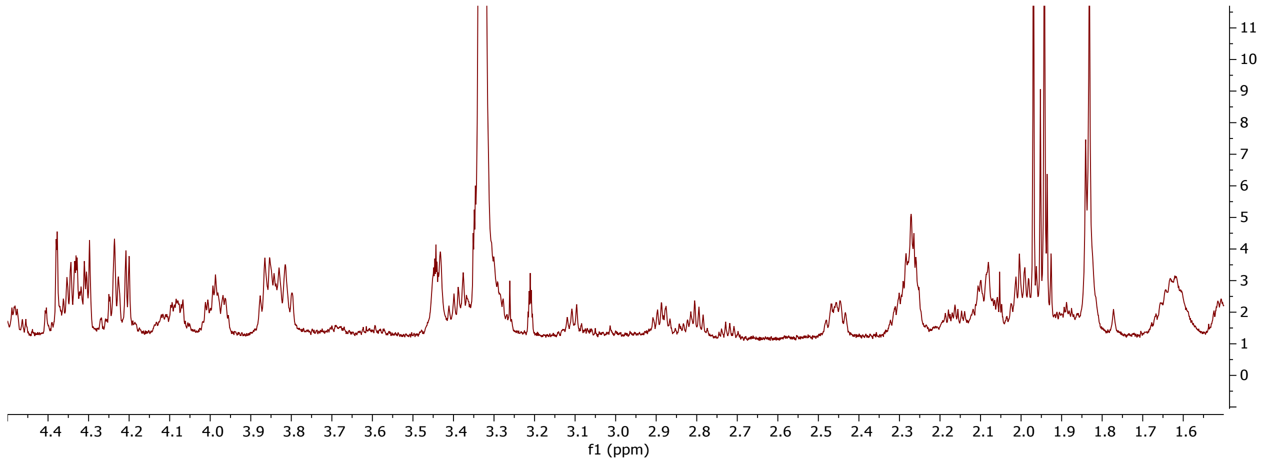

Figure S4. ^{1}H NMR spectrum of a crude mixture of **CaspoCy5-A** and unfunctionalised Caspofungin in CD_3OD in the key 1.5-4.5 ppm range. The spectrum shows the multiplet at 2.94 – 2.76 (caspofungin) shift to 2.85 – 2.68 (CaspoCy5-A).

Table S1. Assignments of the ^{1}H and ^{15}N NMR signals (CD_3OD) related to the amine sidechains of caspofungin derivatives functionalized on amine N^a. *n.d.*: not determined.

	**Chemical shift (ppm)**			
	**Caspofungin**	**Fmoc-N^a-Caspo**	**CaspoMero-A**	**CaspoCy5-A**
**N^a**	25.3	79.8	*n.d.*	*n.d.*
**H^1**	2.94 – 2.76 (m)	2.73 (ddt, *J* = 74.1, 12.1, 6.3 Hz)	2.84 – 2.64 (m)	2.85 – 2.68 (m)
**H^2**	3.03 – 2.94 (m)	3.28 – 3.20 (m)	3.41 – 3.29 (m)	3.39 – 3.31 (m)
**N^c**	42.8	47.0	*n.d.*	*n.d.*
**N^b**	29.4	29.5	*n.d.*	*n.d.*
**H^3**	3.09 – 3.03 (m)	3.12 – 3.02 (m)	3.13 – 3.01 (m)	3.18 – 3.03 (m)
**H^4**	2.02 – 1.93 (m), 1.89 – 1.77 (m)	2.12 – 2.01 (m), 1.89 – 1.80 (m)	2.14 – 2.00 (m), 1.88 – 1.82 (m)	2.12 – 2.04 (m), 1.92 – 1.81 (m)
**H^5**	4.05 (dddd, J = 18.3, 10.2, 5.7, 2.6 Hz)	4.10 – 4.02 (m)	4.10 (t, J = 8.6 Hz)	4.11 – 4.03 (m)

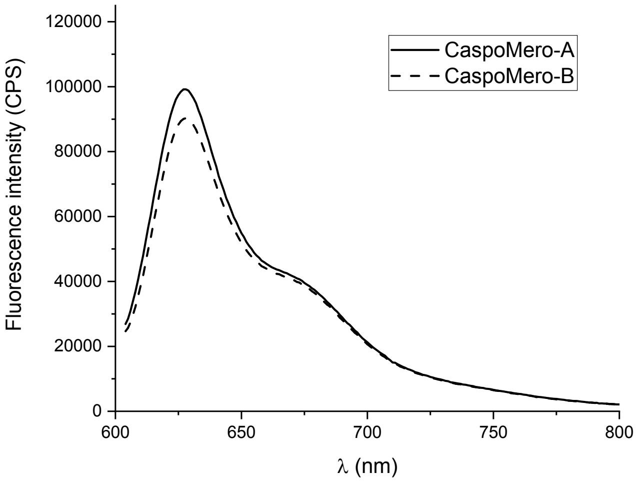

Figure S5. Comparison of the emission properties of **CaspoMero-A** and **-B** (10 µM) in H_2O:DMSO (75/25, *v/v*).

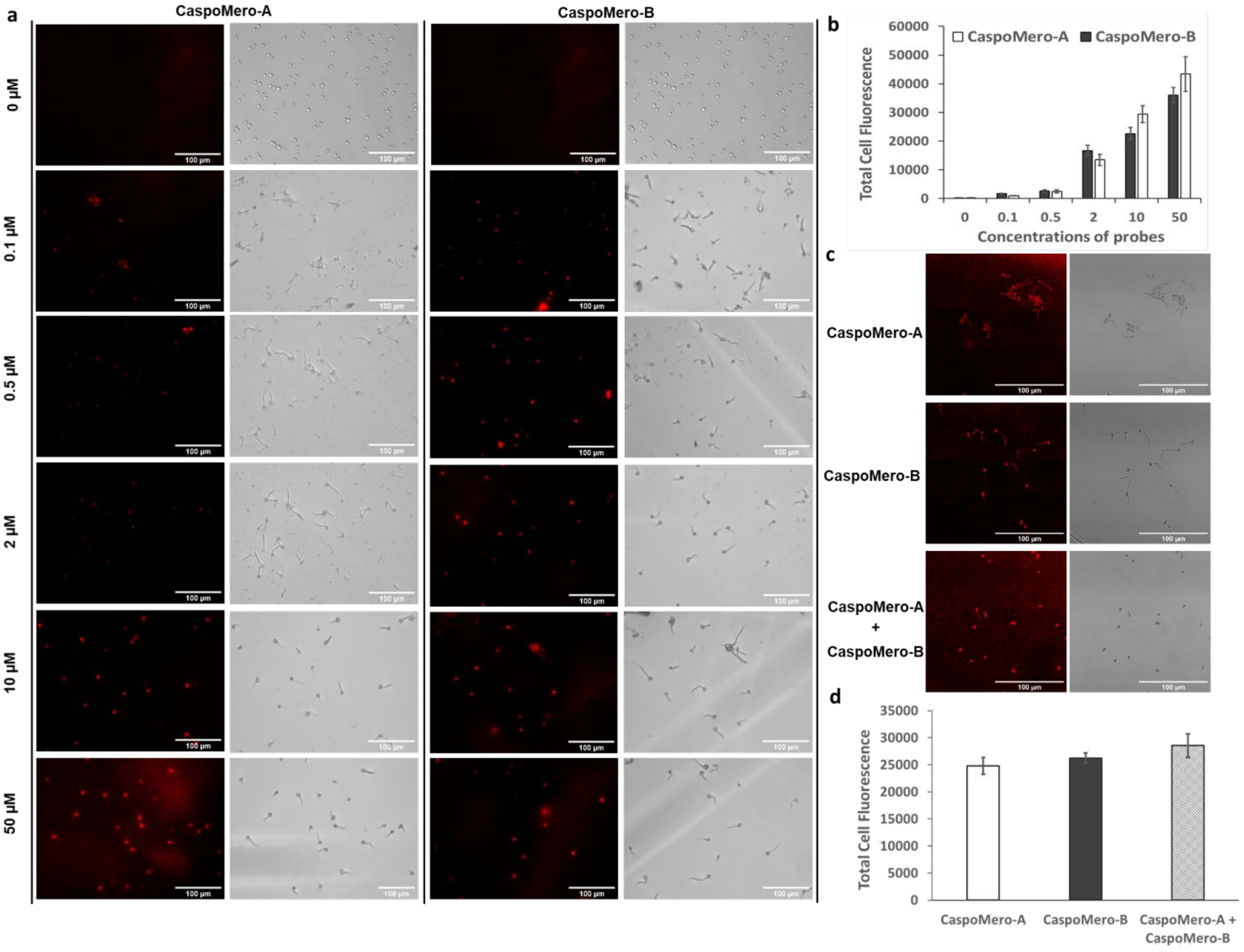

Figure S6. (a) Wash-free labelling of *C. albicans* (10^5 cfu/mL) immobilized onto poly-d-lysine coated wells of

Ibidi µ-Slide 15-Well slides at different concentrations of (a) CaspoMero-A and CaspoMero-B with EVOS M5000 benchtop fluorescent microscope TRITC filter, (b) Quantification of fluorescence intensity was done using ImageJ (NIH) by measuring he mean intensity of 20 fluorescent spots and subtracting the mean of 10 background spots. Standard errors are represented by bars (n=20). (c) and (d) individual isomers and mixed isomers with equimolar ratios (2 µM) show no significant difference in labelling intensities of unwashed *C. albicans* (10^5 cfu/mL) immobilized onto poly-d-lysine coated wells of Ibidi µ-Slide 15-Well slides (imaged through a Leica TCS SP5 laser-scanning confocal microscope). Left column: Fluorescent images, right column: Brightfield images obtained with a transmitted light photomultiplier tube detector. Scale bars 100 µm.

Table S2. Imaging performance metrics for *C. albicans* labelling with the caspofungin probes. Signal-to-background ratio was calculated as a ratio of the total cell fluorescence of labelled cells (treated) to that of unlabelled cells (blank).

Probe	LOD (µM)[a]	LOQ (µM)[b]	Linear quantification range (µM)[c]	Treated vs untreated fold increase at standard working concentration (2 µM)[d]
**CaspoMero-A**	0.01	0.04	0.04 – 2.0	1411
**CaspoMero-B**	0.01	0.03	0.03 – 2.0	1563
**CaspoCy5-A**	1.9	5.8	5.8 - 50	9.1

a) Limit of detection (LOD) and b) limit of quantification (LOQ) calculated using the standard calibration curve definitions derived from the initial linear segments (see below). c) defined as the range from LOQ to the maximum value of the linear range. d) Signal-to-background (S/B) ratio was calculated as a ratio of the total cell fluorescence of labelled cells (treated) to that of unlabelled cells (blank).

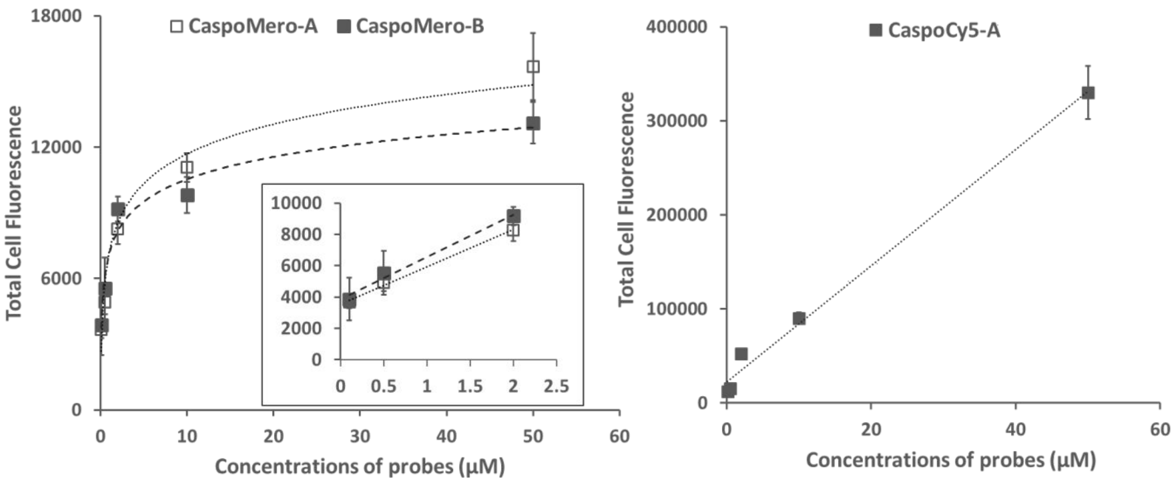

Figure S7. Calibration curves for the fluorescence signal of CaspoMero-A and -B and CaspoCy5-A (0.1 – 50 µM) in *C. albicans*. The fluorescence signal of merocyanine probes followed a logarithmic function (inset shows the linear range), whilst CaspoCy5-A showed a linear correlation for the concentration range tested. LOD and LOQs were calculated based on the linear range of the curve close to expected detection limits.

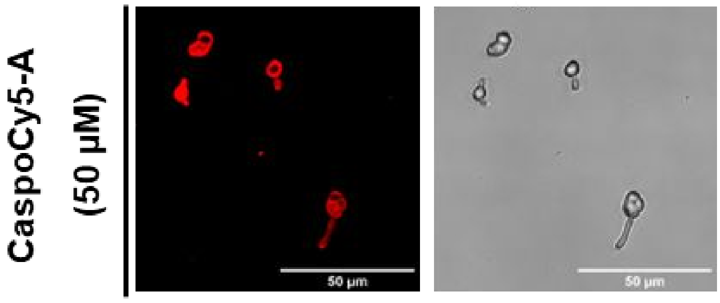

Figure S8. Wash-free labelling of *C. albicans* pseudohyphae immobilized onto poly-d-lysine coated wells of Ibidi µ-Slide 15-Well slides with CaspoCy5-A (50 µM) under a Leica TCS SP5 laser-scanning confocal microscope (λex = 650 nm and λem = 690 nm).

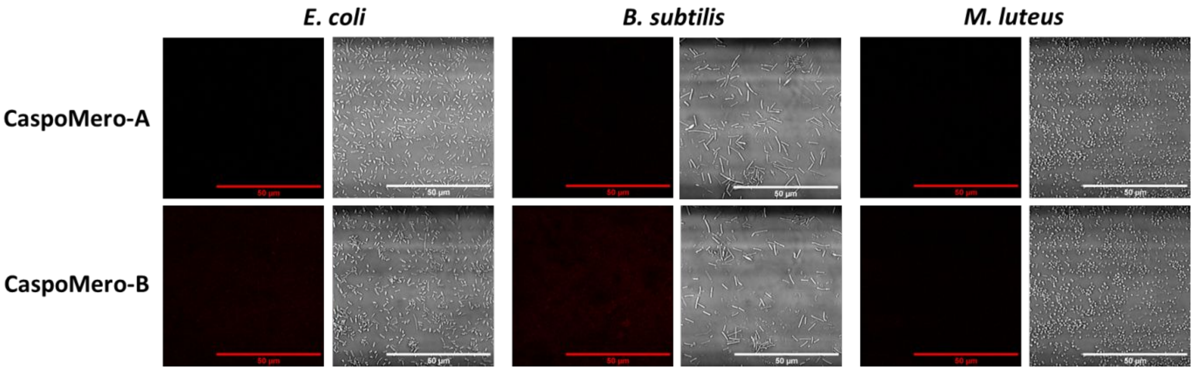

Figure S9. Absence of labelling of Gram-negative bacteria *E. coli* and Gram-positive bacteria *B. subtilis* and *M. luteus* (10^7 cfu/mL) immobilized onto poly-d-lysine coated wells of Ibidi µ-Slide 15-Well slides incubated with CaspoMero-A and CaspoMero-B (5 µM). Images were obtained using a Leica TCS SP5 microscope with λex = 594 nm and λem = 630 nm. Left columns: Fluorescent images, right columns: Brightfield images. Scale bars: 50 µm.

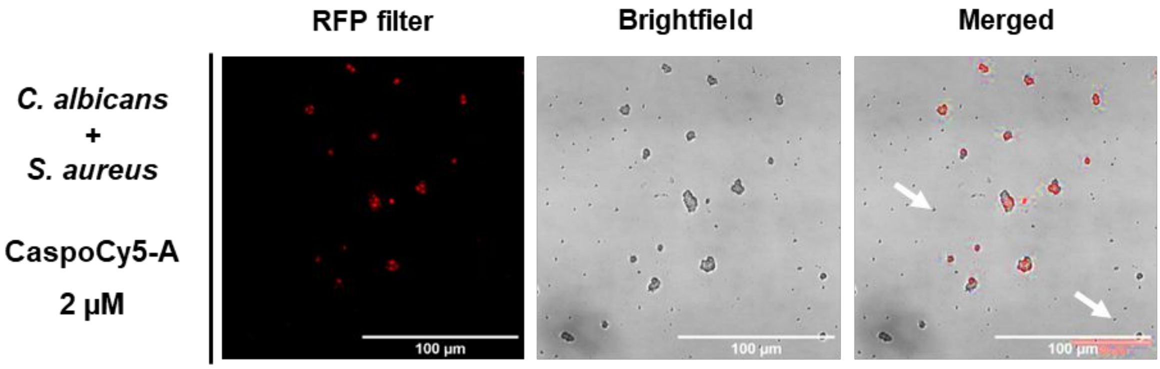

Figure S10. CaspoCy5-A (2 µM) specifically labelling fungal cells in a co-culture of *C. albicans* (10^5 cfu/mL) and the bacteria *S. aureus* (10^8 cfu/mL, visible as dots marked with white arrows) immobilized onto poly-d-lysine coated wells of Ibidi µ-Slide 15-Well slides, using a Leica TCS SP5 laser-scanning confocal microscope (λex = 650 nm and λem = 690 nm). Scale bars: 50 µm.

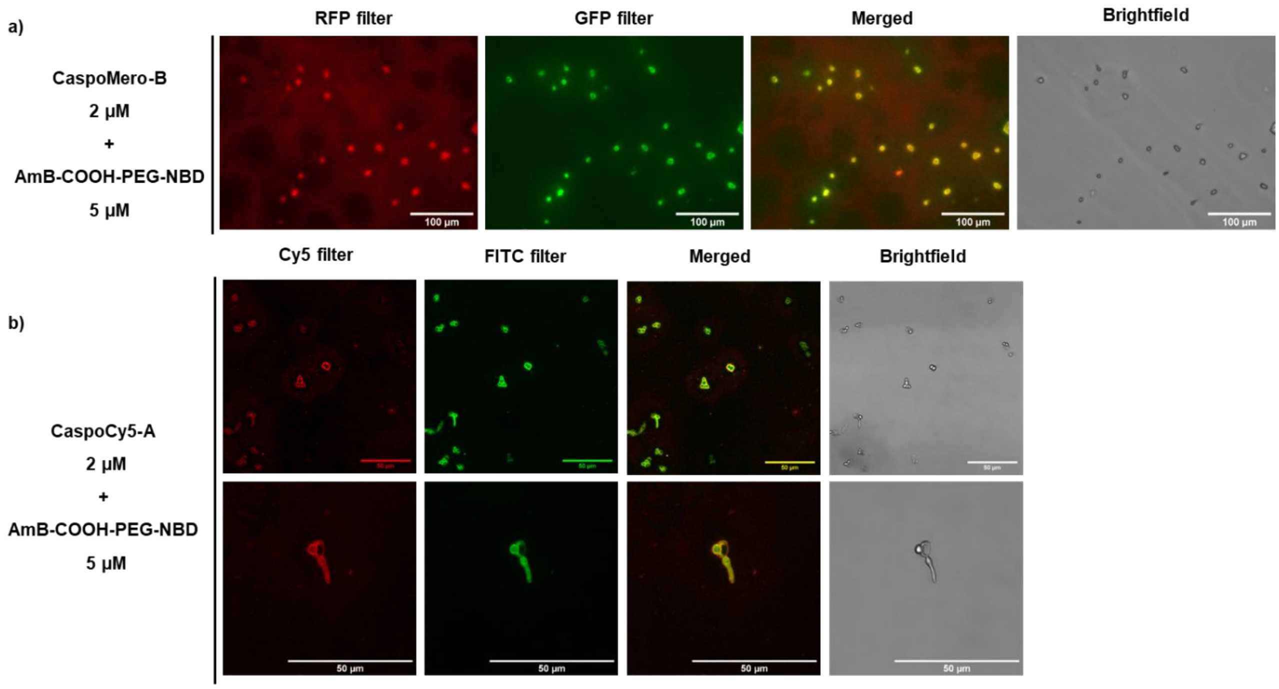

Figure S11. (a) Co-labelling of *C. albicans* (10^5 cfu/mL) immobilized onto poly-d-lysine coated wells of Ibidi µ-Slide 15-Well slides using two different fungal probes CaspoMero-B and AmB-PEG-NBD with EVOS M5000 benchtop fluorescent microscope TRITC and GFP filters (Scale bars: 100 µm), and (b) CaspoCy5-A and AmB-PEG-NBD with Leica TCS SP5 laser-scanning confocal microscope with Cy5 (λex = 650 nm and λem = 690 nm) and FITC filters (λex = 488 nm and λem = 520 nm) (Scale bars: 50 µm).

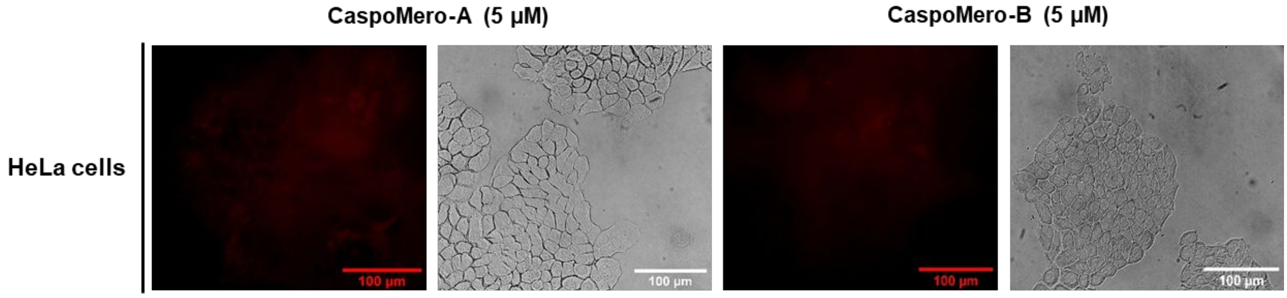

Figure S12. HeLa cells immobilized onto poly-d-lysine coated wells of Ibidi µ-Slide 15-Well slides not labelled with CaspoMero-A (5 µM) and CaspoMero-B (5 µM), captured through the eyepiece of a Zeiss 800 AiryScan microscope (λex = 594 nm and λem = 630 nm). Brightfield images obtained with a transmitted light photomultiplier tube detector. Scale bars: 100 µm.

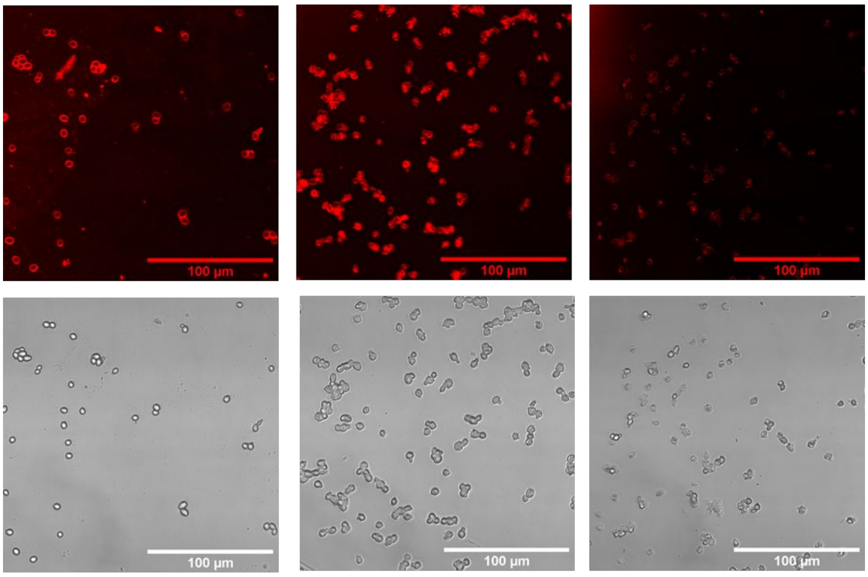

Figure S13. *C. albicans* (10^5 cfu/mL) immobilized onto poly-d-lysine coated wells of Ibidi µ-Slide 15-Well slides treated with CaspoCy5-A for 2, 48 and 72 hours. Gradual disintegration of cells is observed (left: intact cells, middle: disintegrated cell, right: heavily disintegrated). Images were obtained using a Leica TCS SP5 laser-scanning confocal microscope with λex = 594 nm and λem = 630 nm. Top row: Fluorescent images, bottom row: Brightfield images with. Scale bars 100 µm.

II. Chemical synthesis

1. Materials and methods

All air and moisture sensitive manipulations were carried out using standard techniques, with oven-dried reaction vessels, anhydrous solvents, and under nitrogen atmosphere. Extraction and column chromatography solvents were purchased in anhydrous form, and used as received. All reagents were purchased from Fisher Scientific, Aldrich or Fluorochem and used without further purification unless indicated otherwise. Thin layer chromatography (TLC) was performed on Merck silica gel 60 F254 aluminium plaques, and column chromatography was performed on Macherey-Nagel silica gel 60 (40-63 µm). Microwave-assisted reactions were performed on a Biotage Initiator 2.0.

^{1}H, ^{13}C and ^{15}N NMR spectra were recorded on a Bruker AVA 500 spectrometer at 500 MHz and 126 MHz, on a Bruker AVA 600 spectrometer at 600 MHz and 151 MHz, or on a Bruker Avance NEO 800 at 800 MHz, 201 MHz and 81 MHz respectively. Shifts (δ) are given in parts per million with respect to the non-deuterated solvent residual peak for ^{1}H spectra, and relative to the deuterated carbon resonance for ^{13}C and coupling constants (*J*) are given in Hertz. ^{15}N NMR signals between 10 and 140 ppm were recorded using a ^{1}H-^{15}N HMBC sequence.

Analytical reverse-phase high-performance liquid chromatography (RP-HPLC) was performed on an Agilent 1100 system equipped with a Kinetex XB-C18 column (50 × 4.6 mm, 5 µm) with a flow rate of 1 mL/min. Samples were eluted either with a gradient of H_2O/MeCN 95/5 to 5/95, buffered with 0.1% formic acid, over 6 min, then holding at 95% for 3 min, followed by elution at 5% MeCN. Detection was performed with a multiple wavelength detector (MWD) at 254, 282, and 495 and 600 nm, and by an evaporative light scattering (ELSD) detector.

Reverse phase flash chromatography was performed on a Biotage® Isolera One purification system using Biotage® SNAP Ultra C18 columns. Preparative HPLC was performed on an Agilent 1100 system equipped with a Kinetex XB-C18 column (150 × 21.2 mm, 5 µm) with a flow rate of 10 mL/min and eluting with H_2O/MeCN (95/5) to H_2O/MeCN (5/95) all containing 0.1% formic acid, over 25 min, holding at 95% for 3 min, with detection at 550, 600, and 650 nm.

Low resolution electrospray ionization mass spectrometry (ESI-MS) analyses were carried out on an Agilent Technologies LC/MSD Series 1100 quadrupole mass spectrometer (QMS) in ESI mode. HR-MS were obtained by the Mass Spectrometry department of the University of Edinburgh and were performed on a Finnigan MAT 900 XLP high resolution double-focusing mass spectrometer. MALDI spectra were acquired on a Bruker Ultraflextreme MALDI TOF/TOF with a matrix solution of α-cyano-4-hydroxycinnamic acid (10 mg/mL) in H_2O/CH_3CN/TFA (50/50/0.1).

2. Synthetic procedures and characterisation

Functionalisable fluorophores **Mero-COOH** and **Cy5-COOH** were synthesized following literature procedures.[5, 6]

Synthesis of CaspoMero-A and CaspoMero-B from Path A:

To a stirred solution of merocyanine **Mero-COOH** (7.8 mg, 13.6 µmol, 1.1 equiv., synthesised according to the literature[5]) in DMF (0.6 mL) and DIPEA (4.3 µL, 24.7 µmol, 2.0 equiv.) was added HSPyU (5.6 mg, 13.6 µmol, 1.1 eq). The mixture was stirred in the dark for 1 h at 40 °C, and the conversion was monitored by RP-HPLC to confirm the formation of the NHS ester. Caspofungin diacetate (15 mg, 12.4 µmol, 1.0 equiv.) and more DIPEA (4.3 µL, 24.7 µmol, 2.0 equiv.) were then added and the mixture was stirred at r.t. overnight. Solvents were then removed under reduced pressure, and the crude was purified by preparative RP-HPLC (gradient eluent H_2O:MeCN, buffer 0.1% formic acid). Appropriate fractions were combined and freeze-dried to yield the expected tagged peptides as regio-isomers **CaspoMero-A** and **CaspoMero-B** as dark purple powders.

CaspoMero-A:

^{1}H NMR (601 MHz, CD_3OD) δ (selected signals, ppm) 8.15 – 8.06 (t, *J* = 13.0 Hz, 1H, $H_{C=C}$), 7.96 (t, *J* = 7.9 Hz, 2H, $H_{Ar\text{-}Mero}$), 7.89 – 7.79 (m, 4H, $H_{Ar\text{-}Mero}$), 7.21 (d, *J* = 8.7 Hz, 1H, $H_{Ar\text{-}Mero}$), 7.17 (d, *J* = 8.5 Hz, 2H, $H_{Ar\text{-}Casp}$), 7.12 (d, *J* = 8.6 Hz, 1H, $H_{Ar\text{-}Mero}$), 6.85 – 6.79 (m, 1H, $H_{Ar\text{-}Mero}$), 6.77 – 6.72 (d, *J* = 8.2 Hz, 2H, $H_{Ar\text{-}Casp}$), 6.21 (d, *J* = 13.0 Hz, 1H, $H_{C=C}$), 5.01 (d, *J* = 3.2 Hz, 1H), 4.74 (d, *J* = 8.5 Hz, 1H), 4.64 – 4.47 (m), 4.41 (d, *J* = 8.6 Hz, 1H), 4.34 – 4.28 (m, 2H), 4.28 (d, *J* = 1.9 Hz, 1H), 4.20 – 4.14 (m, 2H), 4.10 (t, *J* = 8.6 Hz, 1H, H^5), 4.07 – 3.96 (m, 5H), 3.80 (m, 3H), 3.45 – 3.27 (m), 3.41 – 3.36 (m, 2H, H^2), 3.26 – 3.17 (m, 1H), 3.13 – 3.01 (m, 2H, H^3), 2.84 – 2.64 (m, 2H, H^1), 2.51 – 2.46 (m, 1H), 2.29 – 2.16 (m, 4H), 2.14 – 2.00 (m, 4H, H^4), 1.99 – 1.94 (m, 1H), 1.88 – 1.82 (m, 1H, $H^{4'}$), 1.71 (s, 6H, $H_{Me\text{-}Mero}$).

HR-MS (ESI): m/z = 1646.77000, calcd for $C_{80}H_{116}O_{22}N_{11}S_2$ $[M+H]^+$: 1646.77324. **HPLC (600 nm detection):** t_R = 4.518 min. **UV-Vis (DMSO/H_2O, 75/25):** λ_{abs}^{max} = 599 nm, $\varepsilon^{max} = 3.1 \times 10^4$ M^{-1} cm^{-1}.

CaspoMero-B:

HR-MS (ESI): m/z = 1646.77310, calcd for $C_{80}H_{116}O_{22}N_{11}S_2$ $[M+H]^+$: 1646.77324. **HPLC (600 nm detection):** t_R = 4.575 min (method A). **UV-Vis (DMSO/H_2O, 75/25):** λ_{abs}^{max} = 599 nm, $\varepsilon^{max} = 3.0 \times 10^4$ M^{-1} cm^{-1}.

Synthesis of CaspoCy5-A from path A:

A solution of Sulfonated Cy5-CO_2H (1.1 equiv., synthesized according to the literature[6]) was activated with HSPyU (1.5 equiv.) in DMF (0.3 mL, 0.03 M) and stirred for 2 hours at 40 °C (the formation of the NHS ester

was monitored by analytical HPLC). After completion, the mixture was added to water (1.5 mL) and centrifuged. The resulting purple solid was washed with water, isolated by centrifugation, then purified by preparative RP-HPLC (gradient eluent H_2O:MeCN, buffer 0.1% formic acid). Appropriate fractions were combined and freeze-dried to yield **CaspoCy5-A** as a blue powder.

^{1}H NMR (601 MHz, CD_3OD) δ (selected signals, ppm) 9.26 (d, *J* = 3.0 Hz, 1H, H_{Pyr}), 8.48 (d, *J* = 14.1 Hz, 2H, $H_{C=C}$), 8.37 – 8.28 (m, 5H), 7.95 (d, *J* = 1.9 Hz, 2H, $H_{Ar\text{-}Cy5}$), 7.91 (dd, *J* = 8.3, 1.6 Hz, 2H, $H_{Ar\text{-}Cy5}$), 7.35 (dd, *J* = 8.3, 3.0 Hz, 1H, H_{Pyr}), 7.17 – 7.12 (m, 2H, $H_{Ar\text{-}Casp}$), 6.79 – 6.74 (m, 3H, $H_{Ar\text{-}Casp}$), 5.04 – 4.91 (m, 2H), 4.61 – 4.56 (m), 4.52 – 4.44 (m, 2H), 4.39 – 4.28 (m, 4H), 4.18 – 4.24 (m, 2H), 4.11 – 4.03 (m, 1H, H^5), 4.03 – 3.80 (m, 5H), 3.46 – 3.24 (m), 3.39 – 3.31 (m, 2H, H^2), 3.18 – 3.03 (m, 2H, H^3), 2.85 – 2.68 (m, 2H, H^1), 2.51 – 2.46 (m, 1H), 2.33 – 2.24 (m, 11H), 2.12 – 2.04 (m, 4H, , H^4), 1.99 – 1.94 (m, 2H), 1.92 – 1.81 (m, 6H, $H^{4'}$), 1.97 (s, 3H), 1.94 (s, 3H), 1.84 – 1.82 (m, 6H, $H_{Me\text{-}Cy5}$). **HRMS (MALDI):** m/z = 1779.3112 $[M+MeCN+H]^+$. **HPLC (650 nm detection):** t_R = 4.577 min. **UV-Vis (DMSO):** λ_{abs}^{max} = 650 nm, ε^{max} = 2.9 × 10^4 M^{-1} cm^{-1}.

Synthesis of CaspoCy5-B from Path B:

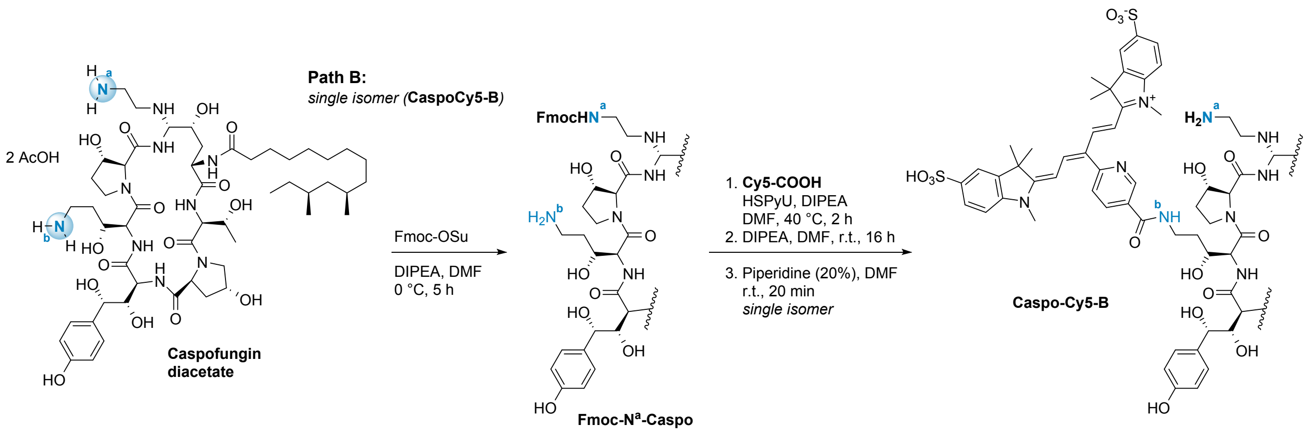

Fmoc-N^a-Caspo:

A stirred solution of caspofungin diacetate (40 mg, 33.0 µmol, 1.0 equiv.) in dry DMF (1.1 mL) and dry DIPEA (59 µL, 330 µmol, 10 equiv.) was placed in an ice bath under argon. Fmoc-OSu (11.1 mg, 33.0 µmol, 1.0 equiv.) was added, and the mixture was stirred at 0-5 °C for 7 h, monitoring the conversion by RP-HPLC. Solvents were then removed under reduced pressure, and the crude was purified by preparative RP-HPLC (gradient eluent H_2O:MeCN, buffer 0.1% formic acid). The Fmoc-protected product was isolated as a white powder (27 mg).

^{1}H NMR (800 MHz, CD_3OD) δ 8.53 (s, 1H), 7.80 (d, *J* = 7.6 Hz, 2H), 7.64 (d, *J* = 7.5 Hz, 2H), 7.39 (t, *J* = 7.4 Hz, 2H), 7.31 (t, *J* = 7.4 Hz, 2H), 7.14 – 7.09 (m, 2H), 6.78 – 6.73 (m, 2H), 4.98 (d, *J* = 3.4 Hz, 1H), 4.92 (d, *J* = 6.3 Hz, 1H), 4.61 (dp, *J* = 6.2, 3.0 Hz, 1H), 4.57 – 4.53 (m, 2H), 4.45 (dd, *J* = 12.4, 5.4 Hz, 1H), 4.35 – 4.28 (m, 5H), 4.23 (dd, *J* = 8.0, 1.8 Hz, 1H), 4.21 – 4.17 (m, 2H), 4.10 – 4.02 (m, 2H, H^5), 3.96 (dd, *J* = 11.1, 3.3 Hz, 1H), 3.84 (td, *J* =

11.3, 4.7 Hz, 2H), 3.78 (dd, *J* = 11.2, 2.0 Hz, 1H), 3.28 – 3.20 (m, 2H, H^2), 3.12 – 3.02 (m, 2H, H^3), 2.73 (ddt, *J* = 74.1, 12.1, 6.3 Hz, 2H, H^1), 2.46 – 2.40 (m, 1H), 2.28 – 2.19 (m, 3H), 2.12 – 2.01 (m, 4H, H^4), 1.95 (dq, *J* = 12.3, 7.1 Hz, 1H), 1.89 – 1.80 (m, 1H, $H^{4'}$), 1.58 (p, *J* = 7.6 Hz, 2H), 1.51 – 1.19 (m, 12H), 1.17 (d, *J* = 6.2 Hz, 3H), 1.13 – 1.04 (m, 1H), 1.01 (td, *J* = 8.0, 4.2 Hz, 1H), 0.92 – 0.80 (m, 12H). **^{13}C NMR (201 MHz, CD_3OD) δ** 176.4, 174.5, 173.5, 173.3, 172.7, 170.2, 168.8, 158.9, 158.6, 145.3, 145.3, 142.6, 133.0, 129.6, 128.8, 128.2, 126.2, 121.0, 116.2, 77.4, 75.6, 75.1, 72.5, 71.3, 69.9, 69.4, 68.3, 67.8, 64.2, 62.8, 58.3, 57.1, 56.4, 56.0, 51.1, 48.4, 47.1, 46.1, 45.9, 41.6, 39.1, 38.5, 38.1, 36.9, 35.3, 34.6, 32.9, 32.9, 31.3, 31.2, 30.7, 30.6, 30.5, 30.4, 30.3, 28.0, 27.1, 20.7, 20.2, 20.0, 11.6. **^{15}N NMR (81 MHz, CD_3OD) δ (selected signals, ppm)** 131.3, 125.9, 124.7, 121.4, 120.3, 115.9, 111.6, 100.6 79.8, 47.0, 29.5. **HR-MS (ESI):** m/z = 1315.7139, calcd for $C_{67}H_{99}O_{17}N_{10}$ [M+H]$^+$: 1315.71842. **HPLC (254 nm detection):** t_R = 4.314 min.

CaspoCy5-B:

To a stirred solution of cyanine **Cy5-COOH** (7.6 mg, 11.4 µmol, 1.5 equiv.) in DMF (0.4 mL) and DIPEA (2.7 µL, 15.2 µmol, 2.0 equiv.) was added HSPyU (4.7 mg, 11.4 µmol, 1.5 equiv.). The reaction mixture was stirred at 40 °C in the dark until complete conversion of the carboxylic acid (3 h). The NHS ester in DMF was then added to a solution of **Fmoc-N^a-Caspo** (10 mg, 7.60 µmol, 1.0 equiv.) in DMF (0.3 mL) at 0 °C, and additional DIPEA (2.7 µL, 15.2 µmol, 2.0 equiv.) was added. the reaction mixture was allowed to warm up to room temperature overnight. The reaction was monitored with LC-MS to show the disappearance of **Fmoc-N^a-Caspo** and the formation of the desired coupling product. A solution of piperidine (10% in DMF, 0.6 mL) was then added and the reaction mixture was stirred at room temperature for 1 h. The mixture was then concentrated under reduced pressure, and the crude purified by prep RP-HPLC (gradient eluent H_2O:MeCN, buffer 0.1% formic acid). Appropriate fractions were combined and freeze-dried to yield **CaspoCy5-B** as a blue powder.

HR-MS (ESI): m/z = 1738.8127, calcd for $C_{85}H_{119}O_{13}N_{22}S_2$ [M+H]$^+$: 1738.8107. **HRMS (MALDI):** m/z = 1779.2670 [M+MeCN+H]$^+$. **HPLC (600 nm detection):** t_R = 4.030 min.

III. Photophysical experiments

All photophysical studies were performed with freshly prepared air-equilibrated solutions at room temperature (298 K). UV/Vis absorption spectra of ~10^{-5} M solutions were recorded on an Agilent 8453 spectrophotometer. Steady-state fluorescence measurements were performed on dilute solutions (ca. 10^{-6} M, optical density ≤ 0.1) contained in standard l = 1 cm quartz cuvettes using a Shimadzu RF-6000 spectrofluorometer. The emission spectra were corrected for the wavelength-sensitivity of the detection unit, obtained, for each compound, under excitation at the wavelength of the absorption maximum. Fluorescence

quantum yields were measured according to literature procedures[1, 2] using Rhodamine-6G (Φ_f^{ref} = 0.94 in EtOH, λ_{exc} = 488 nm) or Cresyl Violet (Φ_f^{ref} = 0.54 in MeOH, λ_{exc} = 570 nm) as reference.[3] The emission quantum yield values Φ_f derived from these measurements were calculated with the following equation taking into account the refractive index (n), the absorbance (A), and the integral of the emission $I_f(\lambda_{exc},\lambda_f)$ of the novel sample (superscript *S*) relative to the reference (superscript *ref*):

$$\Phi_f^S = \Phi_f^{ref} \times \left(\frac{n^S}{n^{ref}}\right)^2 \times \frac{1-10^{-A^{ref}(\lambda_{exc})}}{1-10^{-A^S(\lambda_{exc})}} \times \frac{\int_0^\infty I_f^S(\lambda_{exc},\lambda_f)\,d\lambda_f}{\int_0^\infty I_f^{ref}(\lambda_{exc},\lambda_f)\,d\lambda_f}$$

IV. Biology

Preparation of solutions

Concentrated stock solutions of the following compounds were prepared from powder in phosphate buffered saline (PBS). All compounds were stored at -20 ˚C in the dark.

Microbial cultures

Microbial strains used were *Candida albicans* [American Type Culture Collection (ATCC) SC5314], *Candida auris* (ATCC SC5314 78), *Malassezia furfur* (ATCC 14521) and *Staphylococcus aureus* (ATCC 23235). HeLa cells (Western General Hospital) were used for cross-labelling studies.

Growth of microbial and mammalian cell cultures

Three fungal, 1 bacterial and 1 mammalian cell type were used in this study. Stocks of all strains were stored in glycerol at -80° C. Fungal strains were inoculated into liquid medium - Roswell Park Memorial Institute (RPMI) and Yeast extract Peptone Dextrose (YPD) (ThermoFisher Scientific, Basingstoke, UK) and grown overnight at 30 °C with shaking till exponential phase. Bacteria were grown to exponential phase by inoculating in nutrient broth (ThermoFisher Scientific, Basingstoke, UK) and incubating at 37 °C for 16 h. The overnight cultures were centrifuged and washed twice with phosphate buffered saline (PBS) to harvest the cells. Mammalian cells were grown till confluency in Dulbecco's Modified Eagle's Medium, (Gibco, Fisher Scientific, UK) at 37 °C, 5% CO_2 without shaking, before trypsinization, centrifugation, washing and adding on to well slides.

In cellulo fungal labelling and confocal assessment

Ibidi µ-Slide 15 Well slides (Thistle Scientific, Warwickshire, UK) were coated with 0.1 mg/mL poly-d-lysine at 37°C for 30 min before washing in PBS. Liquid fungal cultures at concentrations 10^5 cfu/mL (30 µL) were added to the wells and allowed to attach to the bottom for 1 hour. For biofilm formation fungal cultures with density 10^6 cfu/mL (200 µL) were seeded into wells and incubated for 48 hours. To detect any non-specific labelling, Gram positive bacteria were seeded at 10^8 cfu/mL (30 µL) in the same well as target fungi. Mammalian cells were adhered at 30% confluency. The wells were washed with PBS to remove unattached cells. Dyes at required concentrations (in PBS with 5% DMSO) were added to the cells and incubated for 2 hours in dark. Labelled cells were imaged with a benchtop EVOS M5000 (Thermofisher Scientific, Basingstoke, UK) microscope with RFP filter settings (575 nm to 640 nm) and a Leica TCS SP5 spinning-disk laser scanning confocal fluorescence microscope (Leica Microsystems, Milton Keynes, UK) with HeNe 594 and 633 laser sources, and TRITC (532 nm – 613 nm) and Cy5 (672 nm – 712 nm) filters. Within each experiment, acquisition parameters were held constant and detector saturation was avoided. On the confocal system, spectral windows were restricted to minimize excitation bleed-through. When Cy5 signals approached detector protection limits, the gain was reduced to maintain linearity.

For co-labelling with two dyes on the same fungal cells (*C. albicans*), an amphotericin-NBD probe[4] (5 µM) and **CaspoMero-B** (2 µM) were added to the inoculated slides. After labelling the image was obtained with FITC filter for the green NBD probe (Excitation wavelength 488 nm) and TRITC for **CaspoMero-B** (Excitation wavelength 594 nm). Images were analysed using ImageJ (NIH, USA).

C. albicans cells were treated with CaspoCy5-A and imaged after 2 hours, 48 hours and 72 hours where gradual disintegration of cell morphology was observed along with change in fluorescence, to study probe retention by the cells.

Fluorescence quantification and analytical metrics

For each probe, *C. albicans* (10^5 cfu/mL) were imaged under identical excitation/emission settings (power, exposure, detector gain). Cell fluorescence was quantified as the mean intensity of selected fungal regions of interest (n = 20 per condition) with background subtraction from cell-free regions (n = 10). Blank is determined from unlabelled cells imaged under identical conditions.

Calibration curves (Figure S7) were plotted as the mean total cell fluorescence accross a range of probe concentrations (0.1 – 50 µM). The initial linear segment was used to determine the slope (k_{lin}). The standard

deviation of the blank (SD_{blank}) was computed across blank fields, and LOD and LOQ were calculated from the standard calibration-curve definitions:

$$LOD = 3.3 \times SD_{blank} / k_{lin}$$

$$LOQ = 10 \times SD_{blank} / k_{lin}$$

The quantification range was defined from the LOQ to the upper bound of the linear portion of the calibration curve. For merocyanine probes, linear fits were verified accross 0.05 – 2.0 µM (Figure S7, inset), while CaspoCy5-A was linear over 2 – 50 µM.

Fold-increase at the standard working concentration (2 µM) is reported as the ratio of mean total cell fluorescence (treated) to the unlabelled cells (mean blank).

V. NMR, MS, and HPLC Data

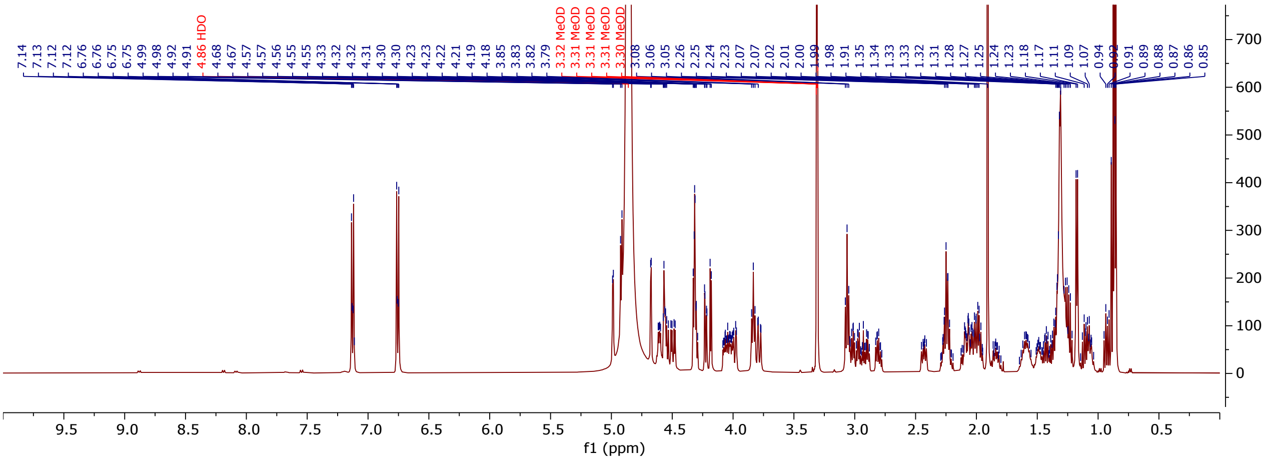

Figure S14. ^1H NMR spectrum (500 MHz) of caspofungin diacetate in CD_3OD.

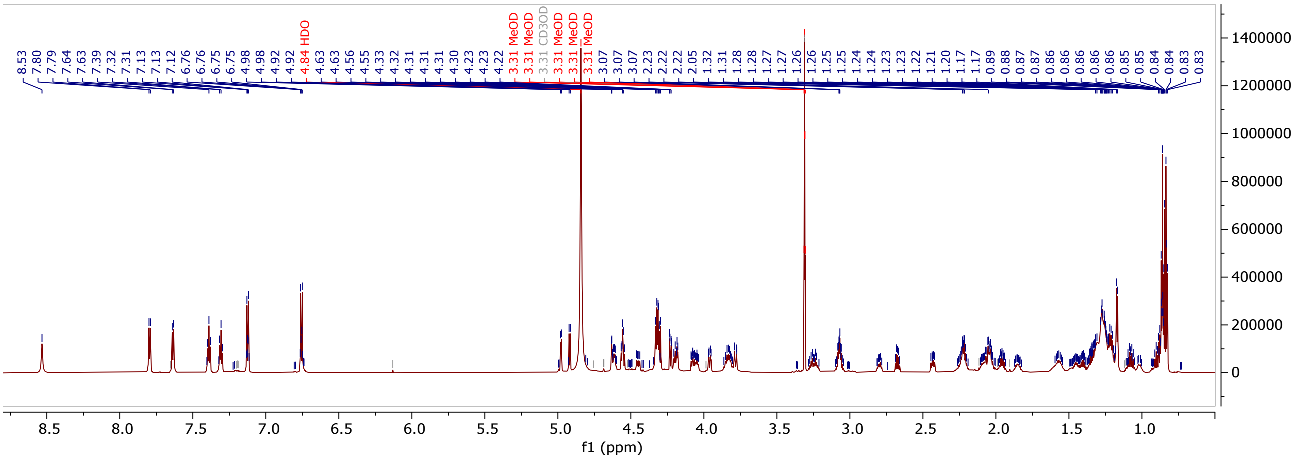

Figure S15. ^1H NMR spectrum (800 MHz) of **Fmoc-N^a-Caspo** in CD_3OD.

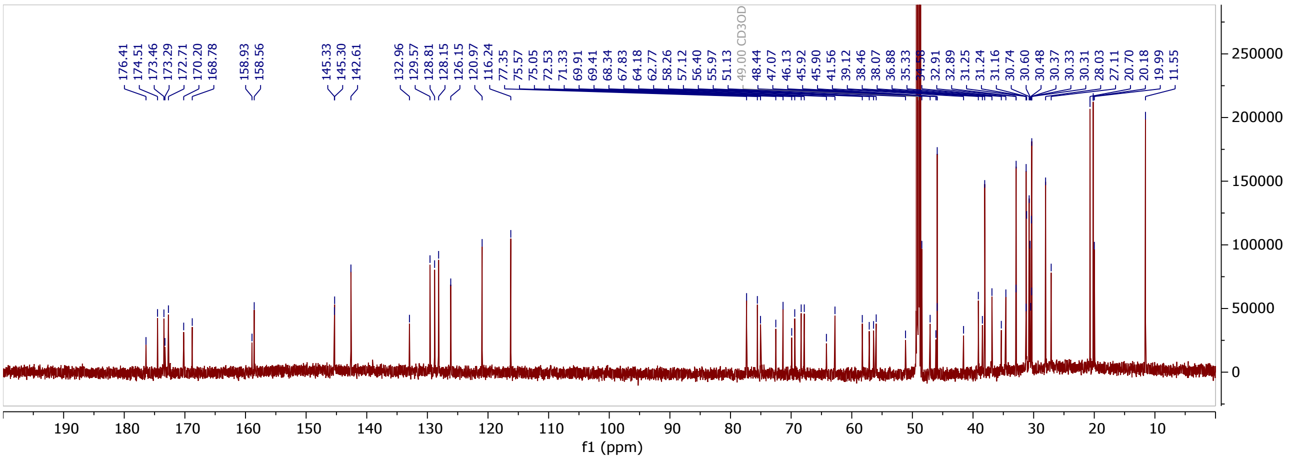

Figure S16. ^{13}C NMR spectrum (201 MHz) of **Fmoc-N^a-Caspo** in CD_3OD.

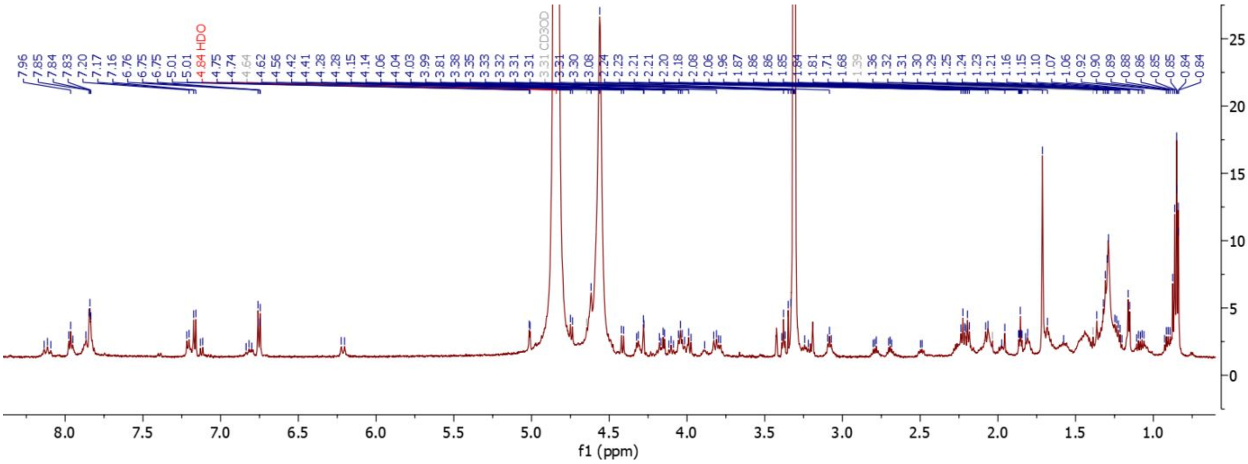

Figure S17. ^{1}H NMR spectrum (601 MHz) of **CaspoMero-A** in CD_3OD.

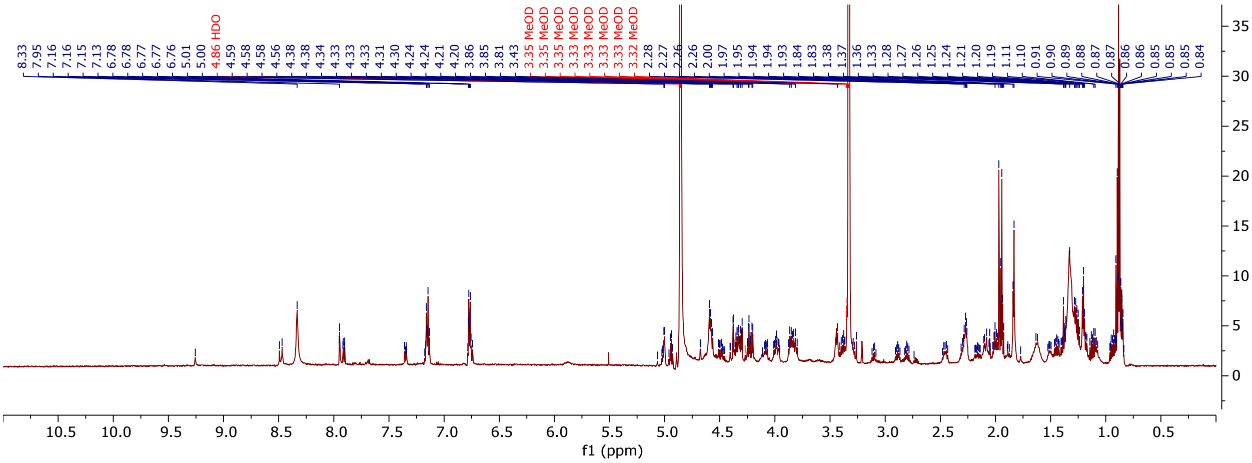

Figure S18. ^{1}H NMR spectrum (601 MHz) of **CaspoCy5-A** in CD_3OD.

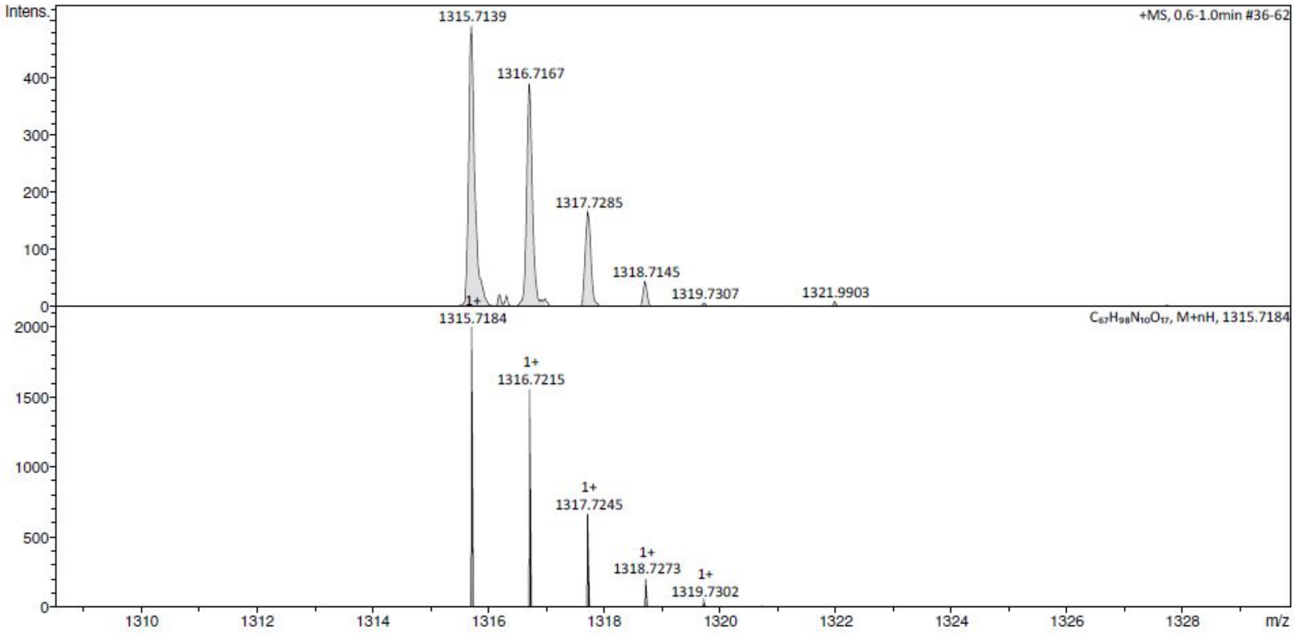

Figure S19. Experimental (top) and theoretical (bottom) HRMS (ESI) spectra for **Fmoc-N^a-Caspo** ([M+H]$^+$).

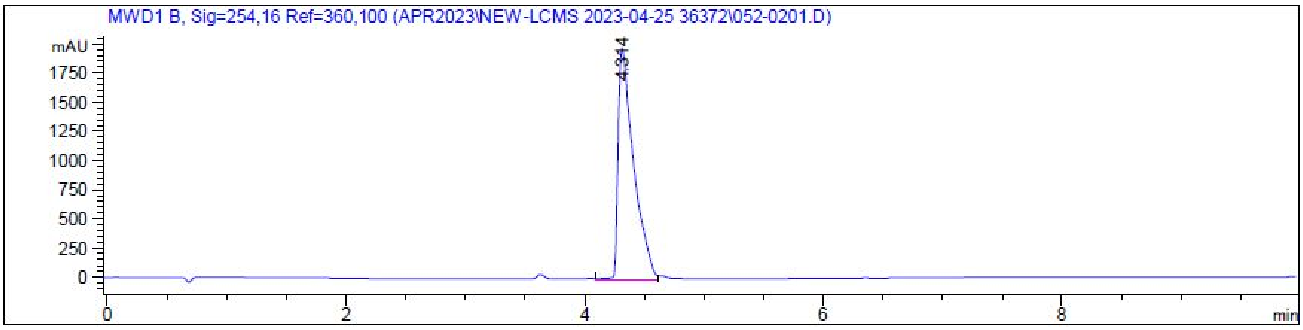

Figure S20. HPLC trace (254 nm detection) for **Fmoc-N^a-Caspo**.

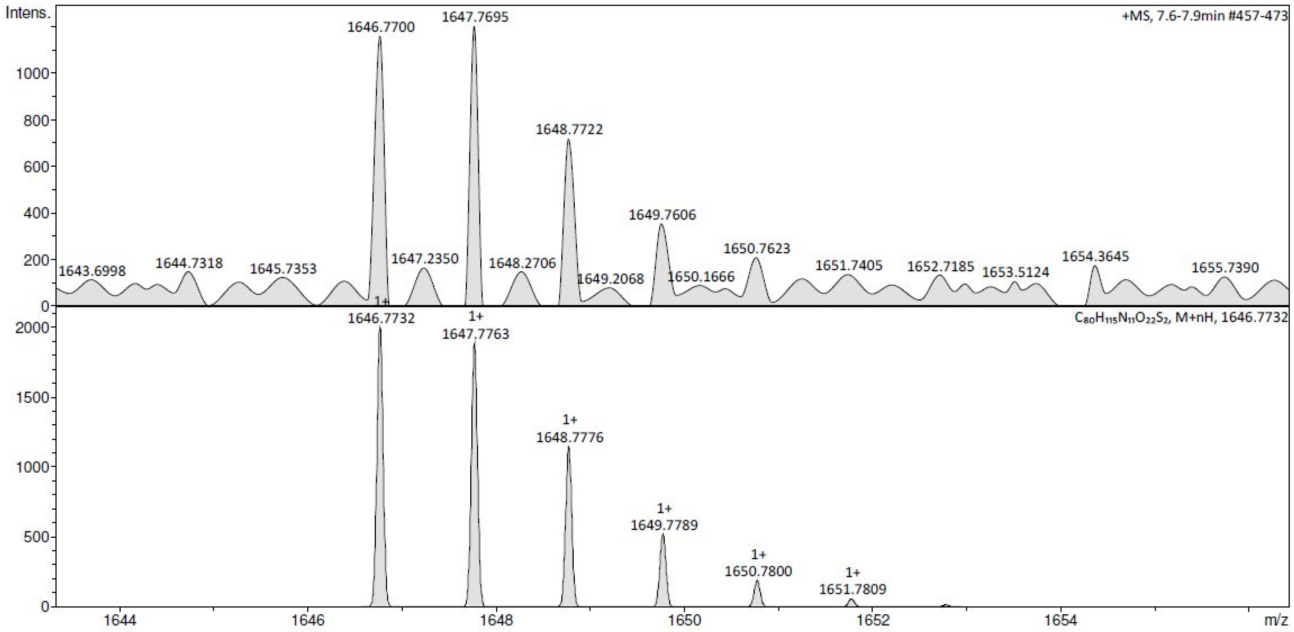

Figure S21. Experimental (top) and theoretical (bottom) HRMS (ESI) spectra for **CaspoMero-A** ([M+H]$^+$).

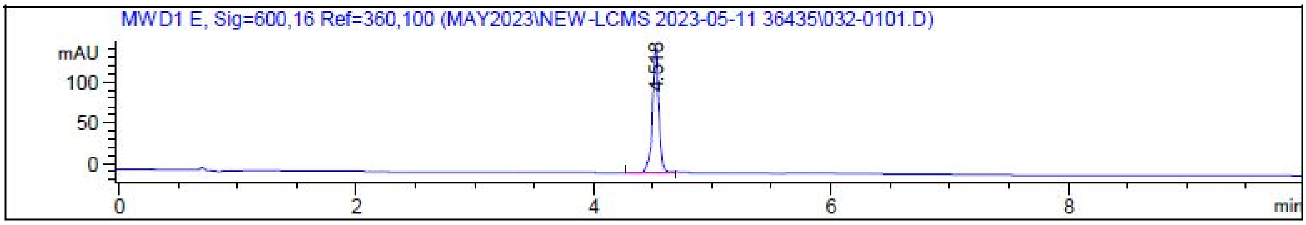

Figure S22. HPLC trace (600 nm detection) for **CaspoMero-A**.

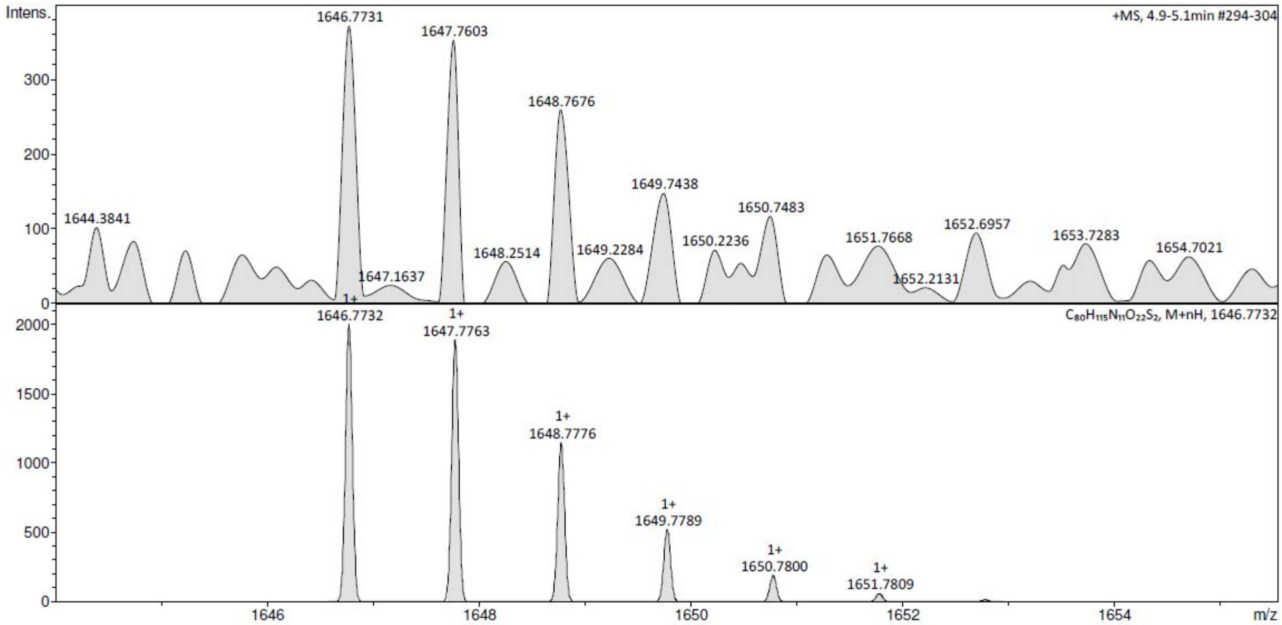

Figure S23. Experimental (top) and theoretical (bottom) HRMS (ESI) spectra for **CaspoMero-B** ([M+H]$^+$).

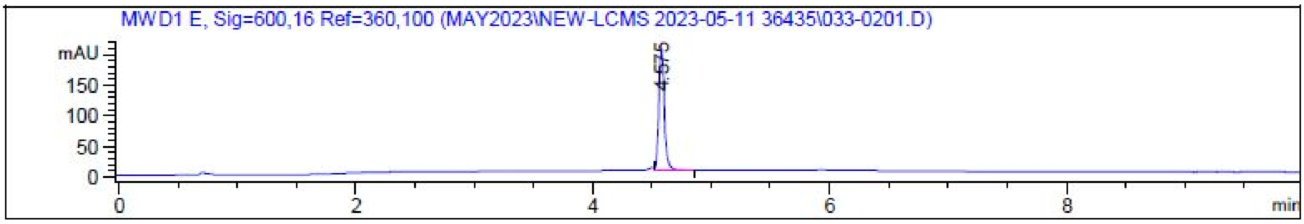

Figure S24. HPLC trace (600 nm detection) for **CaspoMero-B**.

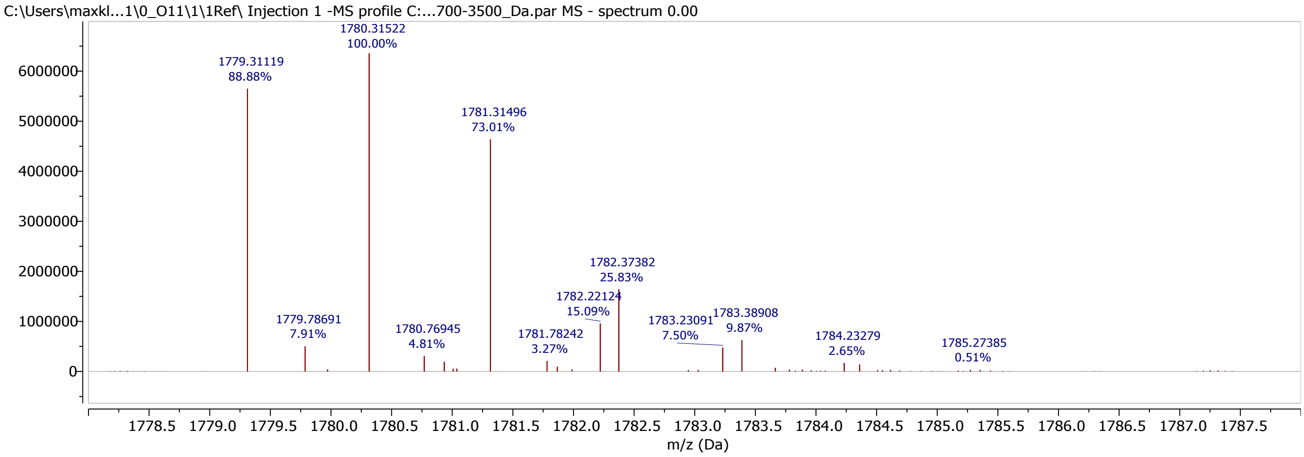

Figure S25. Experimental MS (MALDI) spectrum for **CaspoCy5-A** ([M+MeCN+H]$^{+}$).

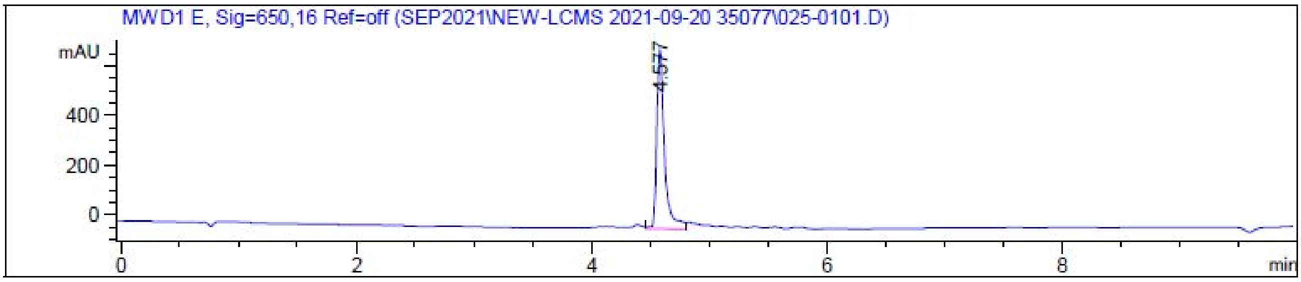

Figure S26. HPLC trace (650 nm detection) for **CaspoCy5-A**.

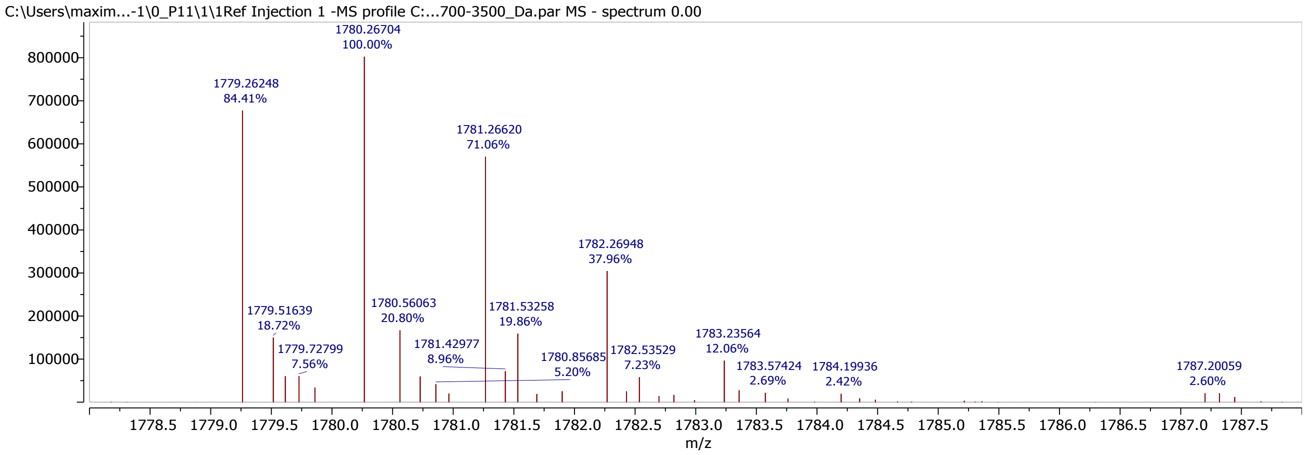

Figure S27. Experimental MS (MALDI) spectrum for **CaspoCy5-B** ([M+MeCN+H]$^+$).

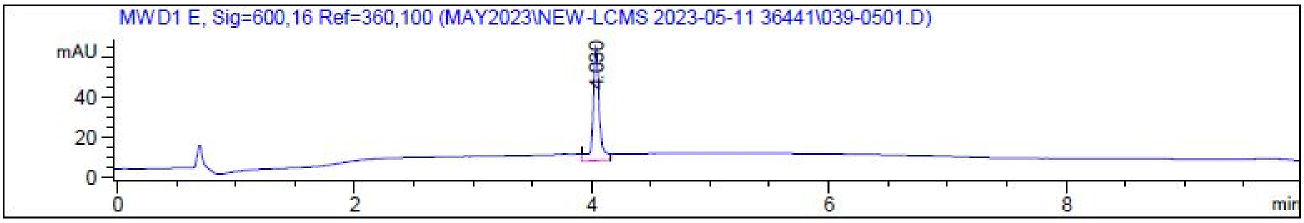

Figure S28. HPLC trace (650 nm detection) for **CaspoCy5-B**.

VI. References

1. U. Resch-Genger and K. Rurack, *Pure Appl. Chem.*, 2013, **85**, 2005-2013.
2. C. Würth, M. Grabolle, J. Pauli, M. Spieles and U. Resch-Genger, *Nat. Protoc.*, 2013, **8**, 1535-1550.
3. A. M. Brouwer, *Pure Appl. Chem.*, 2011, **83**, 2213-2228.
4. A. Baibek, M. Üçüncü, B. Short, G. Ramage, A. Lilienkampf and M. Bradley, *Chemical Communications*, 2021, **57**, 1899-1902.
5. A. Megia-Fernandez, M. Klausen, B. Mills, G. E. Brown, H. McEwan, N. Finlayson, K. Dhaliwal and M. Bradley, *Chemosensors*, 2021, **9**.
6. A. Megia-Fernandez, B. Mills, C. Michels, S. V. Chankeshwara, N. Krstajić, C. Haslett, K. Dhaliwal and M. Bradley, *Organic & Biomolecular Chemistry*, 2018, **16**, 8056-8063.
